# Metabolic Intervention Liposome Boosted Lung Cancer Radio‐Immunotherapy via Hypoxia Amelioration and PD‐L1 Restraint

**DOI:** 10.1002/advs.202207608

**Published:** 2023-04-24

**Authors:** Saijun Wang, Zaigang Zhou, Rui Hu, Mingyue Dong, Xiaobo Zhou, Siyan Ren, Yi Zhang, Chengxun Chen, Ruoyuan Huang, Man Zhu, Wanying Xie, Ling Han, Jianliang Shen, Congying Xie

**Affiliations:** ^1^ Medical and Radiation Oncology Department of the Second Affiliated Hospital of Wenzhou Medical University Wenzhou 325000 China; ^2^ State Key Laboratory of Ophthalmology, Optometry and Vision Science School of Ophthalmology and Optometry, School of Biomedical Engineering Wenzhou Medical University Wenzhou 325027 China; ^3^ Zhejiang Engineering Research Center for Innovation and Application of Intelligent Radiotherapy Technology The Second Affiliated Hospital of Wenzhou Medical University Wenzhou Zhejiang 325000 China; ^4^ Wenzhou Key Laboratory of Basic Science and Translational Research of Radiation Oncology Wenzhou Zhejiang 325000 China; ^5^ Wenzhou Institute University of Chinese Academy of Sciences Wenzhou Zhejiang 325000 China; ^6^ Zhejiang‐Hong Kong Precision Theranostics of Thoracic Tumors Joint Laboratory The Second Affiliated Hospital of Wenzhou Medical University Wenzhou Zhejiang 325000 China

**Keywords:** immunotherapy, lung cancer, oxidative phosphorylation, programmed death ligand‐1, radiotherapy

## Abstract

At present, radiotherapy (RT) still acquires limited success in clinical due to the lessened DNA damage under hypoxia and acquired immune tolerance owing to the amplified programmed death ligand‐1 (PD‐L1) expression. Incredibly, intracellular PD‐L1 expression depression is proven to better sensitize RT by inhibiting DNA damage repair. However, the disability of the clinically used antibodies in disrupting the extracellular PD‐L1function still limits the effectiveness of radio‐immunotherapy. Therefore, better PD‐L1 regulation strategies are still urgently needed to better sensitize radio‐immunotherapy. Hence, for this purpose, TPP‐LND is synthesized by linking mitochondrial‐targeted triphenylphosphine cations (TPP^+^) to the antineoplastic agent lonidamine (LND), which significantly reduces the dose needed for LND to induce effective oxidative phosphorylation inhibition (2 vs 300 µm). Then, TPP‐LND is wrapped with liposomes to form TPP‐LND@Lip nanoparticles. By doing this, TPP‐LND@Lip nanoparticles can sensitize RT by reversing the hypoxic microenvironment of tumors to generate more DNA damage and reducing the expression of PD‐L1 via enhancing the adenosine 5′‐monophosphate‐activated protein kinase activation. As expected, these well‐designed economical TPP‐LND@Lip nanoparticles are more effective than conventional anti‐PD‐L1 antibodies to some extent.

## Graphical abstract

In this study, TPP‐LND@Lip nanoparticles are established to sensitize lung cancer radiotherapy by reversing the hypoxic microenvironment of tumors to generate more DNA damage and reducing the expression of programmed death ligand‐1 (PD‐L1) via enhancing the adenosine 5′‐monophosphate‐activated protein kinase activation. As expected, this well‐designed economical TPP‐LND@Lip nanoparticles are more effective than conventional anti‐PD‐L1 antibodies to some extent.

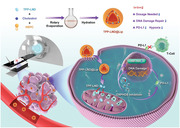

## 1 Introduction

Lung cancer is the most common malignancy and the leading cause of cancer‐related death worldwide, the treatments of which include surgery, radiotherapy (RT), chemotherapy, immunotherapy, and molecular targeted therapy.^[^ ^1^ ^]^ Unfortunately, the majority of advanced lung cancers are intrinsically resistant to various tumor therapies, resulting in cancer progression.^[^ ^2^ ^]^ Among these therapies, radiotherapy is one of the fundamental treatments that has been applied in patients in several stages, and 77% of lung cancer patients have an evidence‐based indication for radiotherapy in the course of their cancer.^[^ ^3^ ^]^ In the past two decades, with the development of precision radiotherapy, the prognosis of patients with lung cancer has improved significantly. Nevertheless, immune resistance induced by higher expressed programmed death ligand‐1 (PD‐L1)after radiotherapy and limited DNA damage due to lung tumor hypoxia still remains one of the main causes of treatment failure after radiotherapy.^[^ ^4^ ^]^ Thus, effective multi‐functional adjuvants that could enhance radiotherapy‐based lung cancer therapy efficacy are still urgently needed.

Programmed death receptor 1 (PD‐1) and its ligand (PD‐L1) are important immune checkpoint pathway molecules. The main function of PD‐L1 located on the cytomembrane to inhibit the antitumor activity of activated T cells. Thus, blocking the membranal PD‐L1/PD‐1 axis is considered an ideal target with great potential for lung tumor immunotherapy.^[^ ^5^ ^]^ Furthermore, it was newly proved that, as a ribonucleic acid (RNA)‐binding protein, intracellular PD‐L1 could regulate the stability of messenger RNA (mRNA) relating to DNA damage genes, thereby increasing the resistance of cells to DNA damage.^[^ ^6^ ^]^ As we all know, radiotherapy induces adaptive upregulation of PD‐L1 expression in lung tumor cells, which may then attenuate the efficacy of anticancer immune responses. Due to this, the administration of anti‐PD‐L1 antibodies further enhances the efficacy of radiotherapy through the cytotoxic T‐cell‐dependent mechanism.^[^ ^7^ ^]^ But in fact, anti‐PD‐L1 monoclonal antibodies have some inevitable disadvantages, such as immunogenicity problems, poor tumor tissue permeability, relatively high cost, and limited efficacy. Besides, most anti‐PD‐L1 antibodies can merely target the PD‐1/PD‐L1 axis on the surface of immune cells and cancer cells rather than intracellular PD‐L1 protein, meaning that these antibodies may not affect the reinforced DNA damage repair (DDR) process mediated by enhanced PD‐L1 expression after radiotherapy.^[^ ^6^, ^8^ ^]^ To sum up, blocking the membranal and intracellular PD‐L1 simultaneously may better enhance the efficacy of radiotherapy in lung cancer therapy.

Recently, studies have shown that a great many cancer cell types rely on mitochondrial respiration and promote tumorigenesis by upregulating oxidative phosphorylation (OXPHOS) activity.^[^ ^9^ ^]^ An increasing number of therapeutic agents have been designed to interfere the mitochondrial biogenesis, directly or otherwise inhibit respiratory chain complexes and disrupt mitochondrial function, such as metformin, IACS‐010759, and lonidamine (LND).^[^ ^10^ ^]^ After OXPHOS inhibition, AMP‐activated protein kinase (AMPK), the energy sensor of nature, is usually activated. Generally, activated AMPK directly phosphorylates the S195 site of PD‐L1 in the lumen of the endoplasmic reticulum (ER), leading to abnormal pruning of mannose during glycosylation, blocking ER‐Golgi translocation, and inducing ER accumulation of PD‐L1. Under the action of SEL1/Hrd1 ubiquitination, PD‐L1 with S195‐p is degraded by the proteasome, the membranal and intracellular expression of which decreased at last.^[^ ^11^ ^]^ Therefore, OXPHOS inhibition may be used as a novel membranal and intracellular PD‐L1 depression strategy to improve the efficacy of radiotherapy in lung cancer treatment.

Aimed at reversing tumor hypoxia to sensitize radiotherapy via enhancing DNA damage, a lot of methods were newly developed, among which the endogenous oxygen generation strategy and enhanced oxygen perfusion strategy were the most widely used.^[^ ^12^ ^]^ Nevertheless, such strategies were nearly all not that effective since only a little oxygen could be delivered or generated, which almost proclaimed the failure of these methods.^[^ ^13^ ^]^ To make up for the defects of these strategies, OXPHOS depression was newly discovered to decrease oxygen consumption to reverse tumor hypoxia. Thus, OXPHOS depression may serve as a better strategy to sensitize tumor radiotherapy by enhancing the DNA damage generation through reversing tumor hypoxia, inhibiting the DNA repair process by reducing the intracellular PD‐L1 expression, and depressing the membranal PD‐L1 expression via disrupting PD‐1/PD‐L1 axis recognition.

**Scheme 1:**
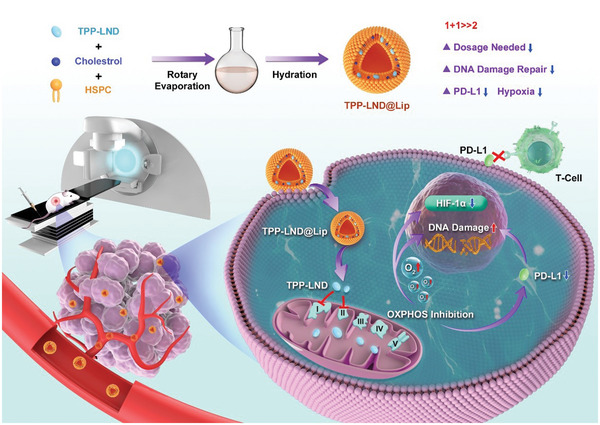
The preparation process of TPP‐LND@Lip nanoparticles and the metabolic intervention mechanism of sensitized radioimmunotherapy by inhibiting PD‐L1 expression and reversing tumor hypoxia.

In this study, the antitumor drug LND, which can inhibit mitochondrial complexes I and II to decrease cellular oxygen consumption, was selected to investigate the possibility of using OXPHOS inhibitors to sensitize radiotherapy by depressing PD‐L1 expression and reversing tumor hypoxia (**Scheme** **1**).^[^ ^14^ ^]^ However, LND diffuses slowly in the cytoplasm due to its high hydrophobicity, and only a minute quantity of LND can be delivered to tumor tissues, especially the mitochondria of cancer cells, so the development of mitochondria‐targeted LND is significantly able to improve its potency.^[^ ^15^ ^]^ To overcome the defects of free LND, we first linked mitochondria‐targeted triphenylphosphine cations (TPP^+^) to LND and then encapsulated them with liposomes to form TPP‐LND@Lip nanoparticles. In this liposome nanoplatform, TPP‐LND therein more effectively activated AMPK by inhibiting OXPHOS compared with LND (2 vs 300 µm), which then enhanced the immune activity of T cells by the down‐regulation of PD‐L1 and reversed the hypoxia of lung cancer tissue at the same time. All in all, TPP‐LND@Lip mediated PD‐L1 downregulation and hypoxia reversion effectively sensitized lung cancer radiotherapy.

## 2 Results

### 2.1 TPP‐LND Activated AMPK and Depressed PD‐L1 Expression by OXPHOS Inhibition In Vitro

Lately, AMPK activation through OXPHOS inhibition is found to be closely related to PD‐L1 downregulation.^[^ ^6c^ ^]^ To prove whether TPP chemically modified OXPHOS inhibition drugs can establish a more ideal PD‐L1 inhibitor, we selected LND as a model drug with OXPHOS inhibition capacity and chemically modified it with TPP for better mitochondria targeting in this study. Generally, TPP‐LND was synthesized by linking TPP with the carboxyl group to the four‐carbon adipose chain, which was regarded as a tumor mitochondria‐targeting molecule, and then the above molecule was bonded together to LND through a condensation reaction (Figure S1, Supporting Information). The obtained molecular weight and structure of TPP‐LND were confirmed by HR‐MS, P‐MRS,^1^H‐NMR, and ^13^C‐NMR (Figures S2–S5, Supporting Information).

**Figure 1:**
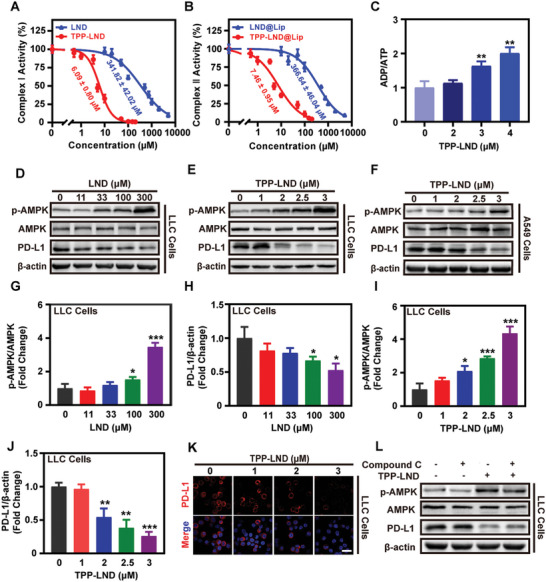
In vitro effects of TPP‐LND on PD‐L1 depression by AMPK activation in lung tumor cells. A,B) Effects of different doses of LND@Lip or TPP‐LND@Lip on the activity of mitochondrial complexes I and II (*n* = 3). C) Detection of ADP/ATP ratio in LLC cells after treatment with TPP‐LND for 12 h (*n* = 3). D–J) Expression of PD‐L1, p‐AMPK, and AMPK protein in LLC and A549 cells by Western Blot assay after treatment with different doses of LND or TPP‐LND for 24 h and further quantification by ImageJ. K) Representative fluorescence images of PD‐L1 expression in LLC cells after being treated with different doses of TPP‐LND, scale bar = 50 µm. L) Detection of PD‐L1, p‐AMPK, and AMPK protein expression in LLC cells by Western Blot assay after treatment with 3 µm TPP‐LND in the presence or absence of AMPK inhibitor (10 µm Compound C) for 24 h (*n* = 3). Data were presented as mean ± SD (*n* = 3). Statistical analysis was performed via the two‐tail Student's *t*‐test. * *p* < 0.05, ** *p* < 0.01, and *** *p* < 0.001.

As mentioned above, by coupling LND with TPP^+^, the effective dose needed by LND to inhibit the mitochondrial complex Ι and II can be greatly reduced.^[^ ^14^, ^15^, ^16^, ^17^ ^]^ To prove that our synthetic TPP‐LND also possesses such an effect, we examined the inhibiting capacity of LND and TPP‐LND on mitochondrial complex I and II, respectively. The analysis verified that TPP‐LND inhibited mitochondrial complex I and II with IC50 of 6.09 ± 0.80 and 7.46 ± 0.95 µm, respectively, while LND was of 341.82 ± 42.02 and 366.64 ± 46.04 µm (**Figure** **1**A,B). The above experimental results suggested that TPP‐LND was about 50 times more potent than LND in inhibiting the function of complexes I and II. Meanwhile, mitochondrial dysfunction would also lead to the reduction of ATP production and ADP consumption, consequently, the ADP/ATP ratio increased with the dose of TPP‐LND increasing (Figure 1C). Thus, TPP‐LND could better inhibit mitochondrial at a lower dosage.

To further confirm whether the above‐mentioned modification can better regulate PD‐L1 expression in vitro, LLC and A549 cells were treated with different concentrations of LND or TPP‐LND. As resulted shown in Figure 1, p‐AMPK was significantly up‐regulated, which then down‐regulated the PD‐L1 protein expression in lung tumor cells with the increase of LND and TPP‐LND concentrations, while TPP‐C4 did not have such effects even at higher concentrations (Figure 1D–J and Figures S6 and S7, Supporting Information). Besides, a similar phenomenon was also observed in LND or TPP‐LND treated MB49 cells (Figure S8A–F, Supporting Information). Interestingly enough, it is worth noting that TPP‐LND could more effectively depress PD‐L1 expression at a very low concentration compared with LND (2 vs 300 µm), which was further confirmed by immunofluorescence staining analysis (Figure 1K and Figure S9, Supporting Information). To investigate whether TPP‐LND mediated PD‐L1 protein downregulation was caused by AMPK activation, LLC cells were treated with 10 µm AMPK inhibitor compound C, showing that the inhibitory effect of 3 µm TPP‐LND on PD‐L1 expression was effectively reversed by compound C (Figure 1L). In conclusion, TPP‐LND was highly likely to inhibit PD‐L1 expression through AMPK activation to some extent by OXPHOS inhibition, and its effective concentration was about 150 times lower than that of LND.

### 2.2 Synthesis and Characterization of TPP‐LND@Lip

**Figure 2:**
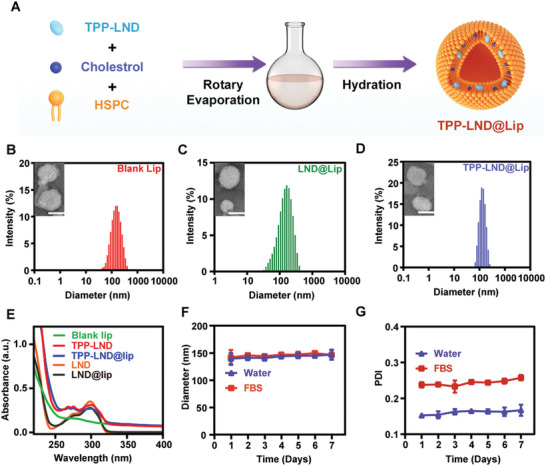
Preparation and characterizations of TPP‐LND@Lip. A) Schematic diagram of the synthesis process of TPP‐LND@Lip nanoparticles. B–D) Hydrodynamic diameters of Blank Lip, LND@Lip, TPP‐LND@Lip, and TEM images of these liposomes. Scale bar = 100 nm. E) Absorption spectra of LND, LND@Lip, TPP‐LND, and TPP‐LND@Lip. F,G) Changes of hydrodynamic diameter and polydispersity index of TPP‐LND@Lip for 7 days in double distilled water and FBS.

Subsequently, TPP‐LND@Lip nanoparticle was prepared by using clinically used liposomes as TPP‐LND carriers through the membrane dispersion method (**Figure** **2**A). Transmission electron microscope images showed the morphology of TPP‐LND@Lip, which was spherical (Figure 2B–D). The particle sizes of Blank Lip, LND@Lip, and TPP‐LND@Lip nanoparticles were 134.2 ± 3.1, 146.8 ± 2.6, and 143.4 ± 2.8 nm, respectively (Figure 2B–D). The zeta potentials of LND@Lip and TPP‐LND@Lip nanoparticles were −5.1 ± 0.4 and 19.9 ± 1.6 mV, respectively (Figure S10, Supporting Information). The content of TPP‐LND in TPP‐LND@Lip nanoparticles was about 87.2% as determined by HPLC and UV–vis absorption spectroscopy (Figure 2E and Figure S11, Supporting Information). Besides, for purpose of predicting the potential application of TPP‐LND@Lip nanoparticles in vivo, the stability of TPP‐LND@Lip nanoparticles was verified by measuring the particle size and polymer dispersion index (PDI) of TPP‐LND@Lip nanoparticles in distilled water and fetal bovine serum (FBS) media. No significant size change was detected for TPP‐LND@Lip nanoparticles in distilled water or FBS for 7 days (Figure 2F,G), suggesting that TPP‐LND@Lip nanoparticles may have ideal drug duration in vivo. Taken together, the results above indicated that TPP‐LND@Lip nanoparticles had the advantages of simple and economical synthesis, excellent stability, and suitable particle size.

### 2.3 TPP‐LND@Lip Up‐Regulated p‐AMPK Expression and Induced Cell Death In Vitro

**Figure 3:**
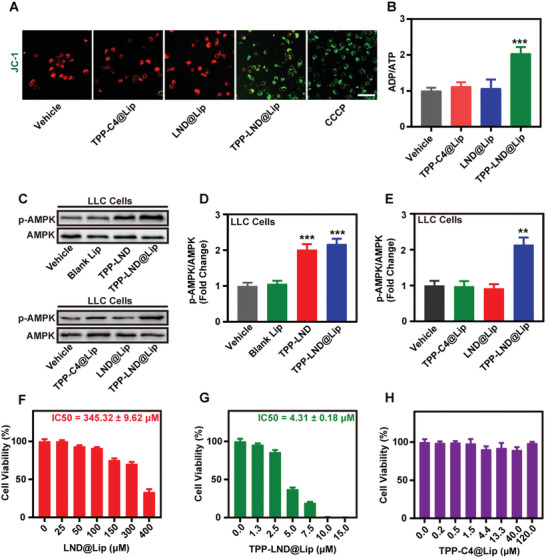
TPP‐LND@Lip induced mitochondria dysfunction and cytotoxicity of TPP‐LND@Lip in vitro. A) Detection of mitochondrial membrane potential (MMP) by JC‐1 assay kit in LLC cells after treatment with 3 µm TPP‐C4@Lip, LND@Lip, or TPP‐LND@Lip for 8 h, scale bar = 50 µm. B) Detection of ADP/ATP ratio in LLC cells after treatment with 3 µm TPP‐C4@Lip, LND@Lip, or TPP‐LND@Lip for 12 h (*n* = 3). C–E) Expression of p‐AMPK and AMPK protein by Western Blot assay in LLC cells after treatment with 3 µm TPP‐C4@Lip, LND@Lip, or TPP‐LND@Lip for 24 h. F–H) Cytotoxicity of the LLC cells detected by CCK‐8 assay after being treated with LND@Lip, TPP‐LND@Lip, or TPP‐C4@Lip for 24 h. Data were presented as mean ± SD (*n* = 3). Statistical analysis was performed via the two‐tail Student's *t*‐test. * *p* < 0.05, ** *p* < 0.01, and *** *p* < 0.001.

Previous experiments have shown that TPP‐LND was capable of activating AMPK by OXPHOS suppression (Figure 1). Similarly, TPP‐LND encapsulated in liposomes may inhibit mitochondrial OXPHOS at a very low concentration and lead to a drop in mitochondrial membrane potential (MMP). According to the data of the MMP detection using probe JC‐1 reagent, the green fluorescence intensity of JC‐1 monomer in LLC cells treated with TPP‐LND@Lip was significantly stronger than that of TPP‐C4@Lip and LND@Lip at the same dosage (**Figure** **3**A and Figure S12, Supporting Information). As stated before, ATP production and ADP consumption decreased due to mitochondrial dysfunction, which was manifested in the increased ADP/ATP ratio with the treatment of TPP‐LND@Lip, while the TPP‐C4@Lip and LND@Lip with the same dose possessed no such effect (Figure 3B). As we all know, any treatment that leads to ADP/ATP ratio increase would lead to the activation of AMPK with a high probability. Thus, increased ADP/ATP ratio would further promote phosphorylation of AMPK in tumor cells induced by TPP‐LND@Lip, but not by TPP‐C4@Lip or LND@Lip (Figure 3C–E and Figures S8G and S13, Supporting Information).^[^ ^18^ ^]^

In the next place, the tumor cell viability after TPP‐C4@Lip, LND@Lip, or TPP‐LND@Lip treatment was detected with the CCK‐8 kit. As these data indicated, the proliferation of LLC cells and A549 cells were more obviously inhibited after TPP‐LND@Lip treatment, which was only about 1/80 and 1/55 of the dose needed for LND@Lip treated cells, respectively (Figure 3F,G and Figure S14, Supporting Information). Besides, TPP‐C4@Lip showed no such effect (Figure 3H and Figure S14, Supporting Information). Additionally, the cytotoxicity and live/dead staining of MB49 cells further demonstrated the general antitumor effect of TPP‐LND@Lip (Figures S16 and S17, Supporting Information). In a nutshell, in comparison with LND@Lip, a very low concentration of TPP‐LND@Lip nanoparticles already could play a better anti‐tumor effect in vitro by influencing mitochondrial bioenergetics.

### 2.4 TPP‐LND@Lip Inhibited the Expression of PD‐L1 and Promoted the Activity of T Cells to Kill Tumor Cells In Vitro

**Figure 4:**
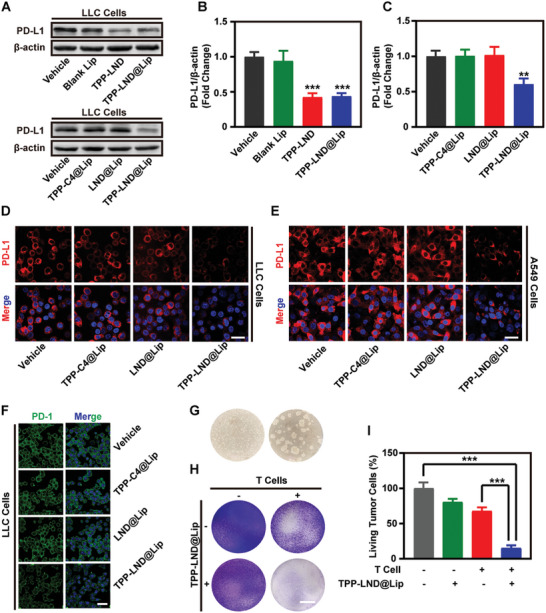
TPP‐LND@Lip suppressed PD‐L1 expression in lung cancer cells in vitro. A–C) Expression of PD‐L1 protein in LLC cells by Western Blot assay after treatment with 3 µm TPP‐C4@Lip, LND@Lip, or TPP‐LND@Lip for 24 h and further quantification by ImageJ. D,E) Representative CLSM images of PD‐L1 immunofluorescence in LLC and A549 cells after being treated with 3 µm TPP‐C4@Lip, LND@Lip, or TPP‐LND@Lip, scale bar = 50 µm. F) Representative fluorescence images of PD‐1 Fc chimera protein in LLC cells after treatment with TPP‐C4@Lip, LND@Lip, or TPP‐LND@Lip, scale bar = 50 µm. G) Morphology of human T cells before and after activation. H,I) Effect of TPP‐LND@Lip on T cell killing in A549 cells after co‐cultured with activated T cell for 48 h and then stained with crystal violet, scale bar = 10 mm. Data were presented as mean ± SD (*n* = 3). Statistical analysis was performed via the two‐tail Student's *t*‐test. * *p* < 0.05, ** *p* < 0.01, and *** *p* < 0.001.

As mentioned above, it was confirmed that the TPP‐LND monomer can inhibit the expression of PD‐L1 by activating AMPK and that TPP‐LND@Lip can phosphorylate AMPK. Thus, TPP‐LND@Lip may also depress PD‐L1 expression as the TPP‐LND monomer did in LLC and A549 tumor cells. The data demonstrated that at the dose of 3 µm, TPP‐LND@Lip but not TPP‐C4@Lip or LND@Lip led to the downregulation of PD‐L1 protein (**Figure** **4**A–E and Figures S8G,  S13,  S18, and S19, Supporting Information). Furthermore, in an effort to explore whether the binding force of PD‐L1 on the tumor membrane and PD‐1 on T cells was affected by TPP‐LND@Lip, the mouse PD‐1(CD279) Fc protein was used.^[^ ^19^ ^]^ As the results of the immunofluorescence staining assay showed, the binding force of PD‐L1 on the tumor membrane with PD‐1 was significantly reduced after treating with TPP‐LND@Lip (Figure 4F and Figure S20, Supporting Information). Generally, the decreased expression of PD‐L1 was conducive to the enhancement of T cell activity. Thus, we also evaluated the effects of TPP‐LND@Lip on the activity of T cells in killing tumor cells (Figure 4G–I). The activated T cells were co‐cultured with A549 cells pretreated with TPP‐LND@Lip, the analysis revealed that TPP‐LND@Lip enhanced the activity of T cells to kill tumor cells (Figure 4H,I). In summary, TPP‐LND@Lip promoted T‐cell‐mediated immunotherapy by reducing the expression of PD‐L1 protein in tumor cells.

### 2.5 The Anti‐Tumor Effect of TPP‐LND@Lip In Vivo

To evaluate the tumor aggregation ability of TPP‐LND@Lip, the in vivo fluorescence imaging in LLC tumor‐bearing mice was used (Figure S21, Supporting Information). More Ce6 fluorescence signals were observed at tumor sites in Ce6@TPP‐LND@Lip treated mice than in free Ce6‐treated mice at 24, 36, and 48 h after administration of Ce6@TPP‐LND@Lip or free Ce6 (Figure S21A, Supporting Information). At 48 h, the mice were sacrificed to collect the major organs and tumors. As results indicated, more Ce6 accumulated in the tumors in the Ce6@TPP‐LND@Lip treated mice compared with the Ce6 group (Figure S21B,C, Supporting Information). Similarly, the fluorescence intensity of Ce6 or ICG in the LLC tumors treated with Ce6@TPP‐LND@Lip or ICG@TPP‐LND@Lip was much higher than that treated with free Ce6 or ICG at 24 and 48 h after administration (Figure S21D–G, Supporting Information). All in all, TPP‐LND@Lip may better accumulate at the site of the tumor.

**Figure 5:**
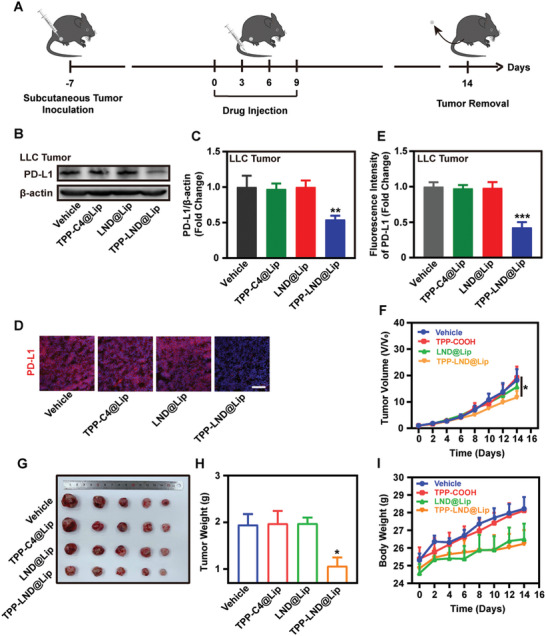
TPP‐LND@Lip suppressed PD‐L1 expression in the LLC tumors and inhibited tumor growth in vivo. A) Schematic diagram of LLC tumor treatment process. B,C) Expression of PD‐L1 protein in LLC model by Western Blot assay after treated with TPP‐C4@Lip, LND@Lip, or TPP‐LND@Lip and further quantification by ImageJ. D,E) Representative fluorescence images of PD‐L1 expression in the LLC model after being treated with TPP‐C4@Lip, LND@Lip, or TPP‐LND@Lip, scale bar = 50 µm. F) LLC tumor growth curve after different treatments for 14 days (*n* = 5). G) Photographs of excised LLC tumors on day 14 after different treatments (*n* = 5). H) Weight of excised LLC tumors on day 14 (*n* = 5). I) Bodyweight changes of LLC tumor‐bearing mice during 14 days (*n* = 5). Data were presented as mean ± SE. Statistical analysis was performed via the two‐tail Student's *t*‐test. * *p* < 0.05, ** *p* < 0.01, and *** *p* < 0.001.

Encouraged by the excellent targeting and antitumor properties demonstrated by TPP‐LND@Lip nanoparticles in vitro, we further evaluated the anticancer effects of these nanoparticles in the LLC tumor model. First, with the aim of evaluating the effect of TPP‐LND@Lip on the expression of PD‐L1 protein in vivo, mice were treated with saline, TPP‐C4@Lip, LND@Lip, or TPP‐LND@Lip, respectively (**Figure** **5**A). Results of western blot analysis confirmed that the expression level of PD‐L1 protein was significantly decreased after TPP‐LND@Lip treatment, which was not observed in other groups (Figure 5B,C) and further confirmed by immunofluorescence staining of LLC tumor sections (Figure 5D,E).

We then tested the antitumor effect of TPP‐LND@Lip in the LLC model. As shown in Figure 5F,G, TPP‐LND@Lip significantly inhibited tumor volume growth, while TPP‐C4@Lip or LND@Lip did not show this ability (Figure S22, Supporting Information). Finally, LLC tumor‐bearing mice were killed on the 14th day, the tumors of which were collected and weighed, and the tumor weight significantly decreased after treatment with TPP‐LND@Lip (Figure 5H). Moreover, there was no significant change in the body weight of mice during the 14 days of treatment (Figure 5I). In general, TPP‐LND@Lip nanoparticles also had excellent anti‐tumor effects in vivo.

### 2.6 TPP‐LND@Lip Intensifies Radiosensitizing Effects by Reversing Tumor Hypoxia

**Figure 6:**
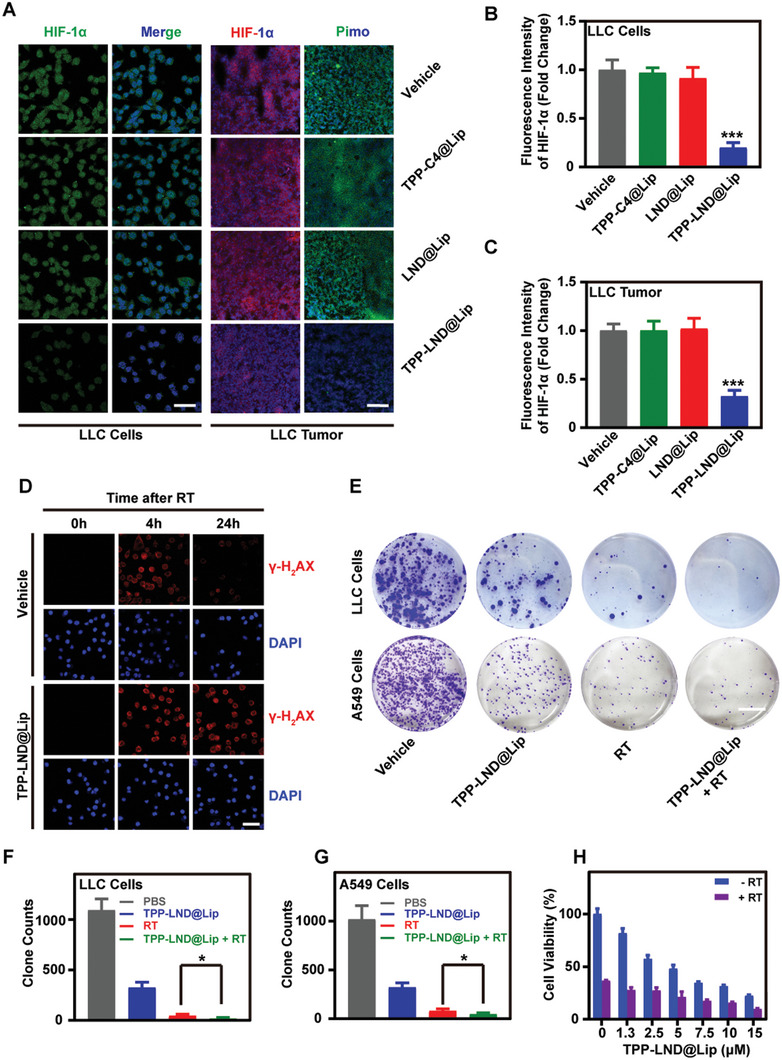
TPP‐LND@Lip reversed tumor hypoxia to increase DNA damage and suppress colony forming. A–C) Representative CLSM images of HIF‐1*α* and pimonidazole in LLC cells and LLC tumor slices, scale bar = 50 µm. D) Representative fluorescence images of *γ*‐H_2_AX in LLC cells with radiotherapy (RT, 6 Gy) and TPP‐LND@Lip (3 µ), scale bar = 50 µm. E–G) Detection of the growth of LLC and A549 cells in the presence or absence of RT (6 Gy) treatment by colony formation assay, scale bar = 10 mm. Quantifications are shown in (F) and (G). H) Cytotoxicity of LLC cells by CCK‐8 assay after treatment with different concentrations of TPP‐LND@Lip in the presence or absence of RT (6 Gy). Data were presented as mean ± SD (*n* = 3). Statistical analysis was performed via the two‐tail Student's *t*‐test. * *p* < 0.05, ** *p* < 0.01, and *** *p* < 0.001.

Studies have shown that LND has the ability to inhibit mitochondrial complex I and II and reduce cell oxygen consumption.^[^ ^20^ ^]^ In previous experiments, TPP‐LND had also been proven to induce mitochondrial dysfunction by inhibiting OXPHOS, and its efficacy was about 50 times that of LND (Figure 1A,B). The OXPHOS system was known to generate energy through the consumption of oxygen, so TPP‐LND@Lip might be able to reverse the hypoxic environment of tumors more effectively.^[^ ^21^ ^]^ To prove this point, the expression of hypoxia‐inducible factor‐1*α*(HIF‐1*α*) in cells was detected by immunofluorescence staining. It was observed that the green fluorescence of HIF‐1*α* protein significantly reduced after treatment with 3 µm of TPP‐LND@Lip, while the same dose of TPP‐C4@Lip or LND@Lip had no such effect (**Figure** **6**A,B). Further, the oxygenation of tumor tissues in vivo was evaluated by two tumor hypoxia markers, HIF‐1*α* and pimonidazole. The expressions of which were significantly decreased 48 h after the injection of TPP‐LND@Lip (Figure 6A–C). Thus, TPP‐LND@Lip could reverse tumor hypoxia both in vitro and in vivo by a metabolic intervention.

It has been shown that DNA free radicals produced by ionizing radiation are usually fixed by O_2_ and then cause DNA damage which may lead to cell death if not repaired, so cells can survive while DNA damage is reduced under the low oxygen conditions.^[^ ^22^ ^]^ With the purpose of evaluating whether the DDR ability of tumor cells was impaired after treatment with TPP‐LND@Lip, dynamic changes of intracellular DNA damage marker *γ*‐H_2_AX were detected by immunofluorescence assay. The data implied that the degradation of *γ*‐H_2_AX was delayed after treatment with TPP‐LND@Lip, while no such delay was observed in the control groups, indicating that the DDR function of tumor cells was impaired and depressed (Figure 6D and Figures S23 and S24, Supporting Information). What is more, ameliorating tumor hypoxia is conducive to the production of more reactive oxygen species (ROS) to improve the efficacy of radiation therapy.^[^ ^22a^ ^]^ Therefore, ROS fluorescent probe DCFH‐DA was used to detect the ROS production capacity of TPP‐LND@Lip and radiotherapy. As shown in Figure S25, Supporting Information, the fluorescence intensity of TPP‐LND@Lip combined with radiotherapy was stronger than that of drugs or radiation alone, suggesting that more ROS could be produced after relieving hypoxia mediated by TPP‐LND@Lip. All in all, TPP‐LND@Lip could impair the process of DDR after radiotherapy.

To further investigate the effect of TPP‐LND@Lip nanoparticles on radiation therapy in vitro, TPP‐LND@Lip was treated together with radiotherapy, and their colony formation capacity was measured further. The results emphasized that the cell growth rate after TPP‐LND@Lip mediated radiation therapy was much slower than that of other groups (Figure 6E–G). Subsequently, the CCK‐8 assay also reached a similar result that LLC cells viability decreased with the increase of TPP‐LND@Lip dose, and the decreasing trend was more obvious after radiation therapy (Figure 6H). However, LND@Lip or TPP‐C4@Lip did not have such a synergistic effect (Figure S26, Supporting Information). Collectively, these findings were consistent with the idea that TPP‐LND@Lip made lung tumor cells more sensitive to radiotherapy by reversing the lack of oxygen in the tumor.

### 2.7 TPP‐LND@Lip Reversed the Amplified PD‐L1 Expression Induced by Radiotherapy

**Figure 7:**
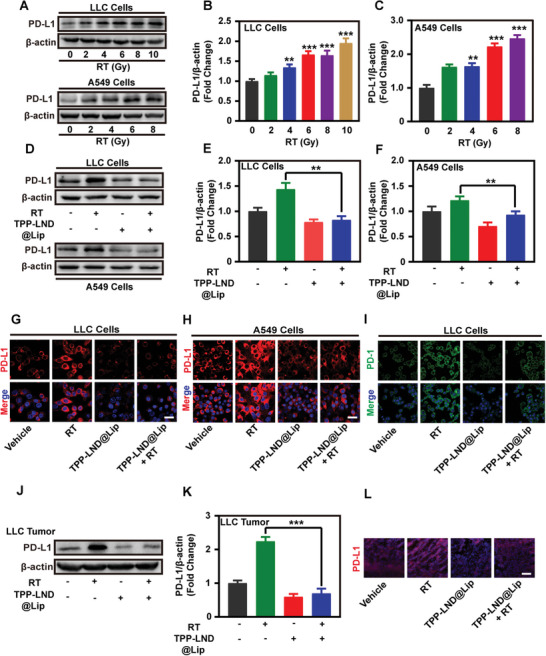
TPP‐LND@Lip suppressed PD‐L1 expression in the presence or absence of radiotherapy. A–C) Expression of PD‐L1 protein in LLC and A549 cells by Western Blot assay after treatment with different doses of RT and further quantification by ImageJ. D–F) PD‐L1 expression in LLC and A549 cells by Western Blot assay in the presence or absence of RT (6 Gy) or TPP‐LND@Lip (3 µm) treatment and further quantification by ImageJ. G,H) Representative CLSM images of PD‐L1 in LLC and A549 cells, scale bar = 50 µm. I) Representative CLSM images of PD‐1 Fc chimera protein in LLC cells with different treatments, scale bar = 50 µm. J,K) PD‐L1 expression in LLC model by Western Blot assay in the presence or absence of RT or TPP‐LND@Lip treatment and further quantification by ImageJ. L) Representative fluorescence images of PD‐L1 in LLC tumor slices, scale bar = 50 µm. Data were presented as mean ± SD (*n* = 3). Statistical analysis was performed via the two‐tail Student's t‐test. * *p* < 0.05, ** *p* < 0.01, and *** *p* < 0.001.

A previous study revealed that intracellular PD‐L1 is able to act as an RNA‐binding protein to protect the mRNA of DDR‐related genes from degradation, thereby increasing tumor resistance to DNA damage and leading to radiotherapy resistance.^[^ ^6a^ ^]^ In addition, radiotherapy can lead to the upregulation of PD‐L1 expression in tumor cells, weakening the effect of immunotherapy.^[^ ^23^ ^]^ Previous studies have confirmed that TPP‐LND@Lip was capable of down‐regulating the expression of PD‐L1 in vivo and in vitro effectively (Figure 4). In order to further prove whether TPP‐LND@Lip could reverse the enhanced expression of PD‐L1 caused by radiotherapy, we treated cells with different doses of radiation, and western blot data verified a dose‐dependent increase in PD‐L1 protein expression (**Figure** **7**A–C). Following this, we treated cells with TPP‐LND@Lip together with radiotherapy. As results showed, the expression of PD‐L1 protein significantly increased in the radiotherapy‐alone group, which then was effectively reversed after the addition of TPP‐LND@Lip (Figure 7D–F). Apart from this, similar conclusions were also drawn by the immunofluorescence staining analysis (Figure 7G,H and Figure S27, Supporting Information). Then, the PD‐1 binding force was correspondingly weakened in the drug‐combined radiotherapy group compared to cells receiving radiotherapy alone as well (Figure 7I and Figure S28, Supporting Information). The western blot and immunofluorescence analysis of the LLC tumor model further confirmed the above conclusions (Figure 7J–L and Figure S29, Supporting Information). These important findings fully demonstrated that TPP‐LND@Lip could effectively reverse the enhancement of PD‐L1 expression caused by radiotherapy.

### 2.8 Antitumor Effect of TPP‐LND@Lip Mediated Radioimmunotherapy In Vivo

**Figure 8:**
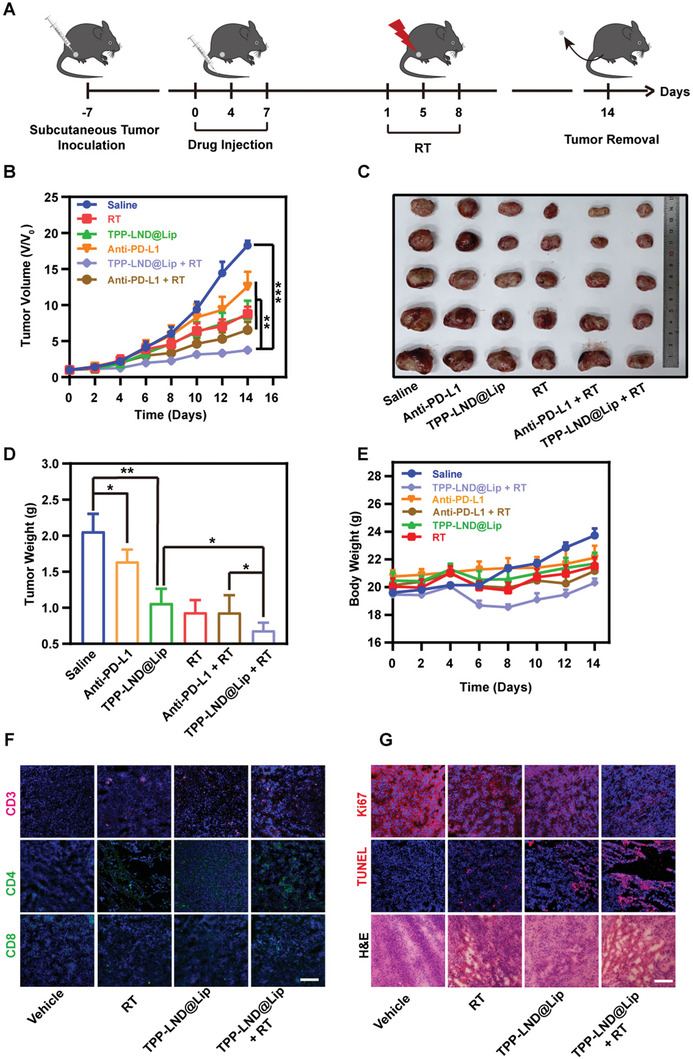
The antitumor effect of TPP‐LND@Lip mediated radiotherapy in vivo. A) Schematic diagram of LLC tumor treatment process. B) LLC tumor growth curve after different treatments for 14 days (*n* = 5). C) Photographs of excised LLC tumors on day 14 after different treatments (*n* = 5). D) Weight of excised LLC tumors on day 14 (*n* = 5). E) Bodyweight changes of LLC tumor‐bearing mice during 14 days (*n* = 5). F) Representative fluorescence images of the CD3^+^ T cells, CD4^+^ T cells, and CD8^+^ T cells in LLC tumor slices, scale bar = 50 µm. G) Immunofluorescence staining of TUNEL, Ki67, and representative H&E images of tumor tissues, scale bar = 50 µm. Data were presented as mean ± SE. Statistical analysis was performed via the two‐tail Student's *t*‐test. * *p* < 0.05, ** *p* < 0.01, and *** *p* < 0.001.

As we proved above, TPP‐LND@Lip has been shown to have excellent antitumor activity in vivo and in vitro. Thus, we further evaluated the radioimmunotherapy sensibilization capacity of TPP‐LND@Lip by using the LLC subcutaneous tumor model (**Figure** **8**A). During 14 days of treatment, the tumor growth rate was significantly reduced treated with TPP‐LND@Lip, PD‐L1 monoclonal antibody, and radiotherapy, and the volume inhibition rate was 53.1 ± 22.3%, 31.3 ± 22.8%, and 51.9 ± 10.8%, respectively. When combining TPP‐LND@Lip or PD‐L1 monoclonal antibody with radiotherapy, tumor growth was almost completely slowed down, with growth inhibition rates of 79.6 ± 5.2% and 64.2 ± 12.2% on day 14, respectively (Figure 8B,C and Figure S30, Supporting Information). Then, LLC tumor‐bearing mice were sacrificed on day 14, all tumors of which were collected and weighed. As results revealed, after treatment with TPP‐LND@Lip or PD‐L1 monoclonal antibody, tumor weight was decreased by 48.4 ± 19.6% and 20.2 ± 16.1%, respectively, compared with the control group (Figure 8D). After treatment with TPP‐LND@Lip combined with radiotherapy, tumor weight was decreased by 66.6 ± 10.4%, while in the PD‐L1 monoclonal antibody with radiotherapy group, tumor weight was only decreased by 54.7 ± 23.6% (Figure 8D). Additionally, no significant changes in body weight were observed in all mice throughout the treatment (Figure 8E).

Encouraged by the exceedingly good capacity of TPP‐LND@Lip in sensitizing radioimmunotherapy in the subcutaneous tumor model, we assessed whether it performed equally well efficacy in the orthotopic lung cancer model established by LLC cells (Figure S31, Supporting Information). As results indicated, the TPP‐LND@Lip+RT group distinctly eliminated the lung tumor burden compared with the other groups, suggesting that TPP‐LND@Lip could enhance the antitumor efficacy of RT (Figure S31B,C, Supporting Information). In addition, TPP‐LND@Lip without RT combination therapy, also more effectively inhibited LLC tumor growth when compared with the PD‐L1 antibody group (Figure S31B,C, Supporting Information). In terms of lung weight, the average weight of mice treated with saline was about 2–3 times heavier than that of the TPP‐LND@Lip + RT group (Figure S31D, Supporting Information). In conclusion, TPP‐LND@Lip, which had the ability to regulate hypoxia and PD‐L1 expression, combined with radiotherapy had the most significant inhibitory effect on local tumor growth in LLC tumor‐bearing mice. Interestingly enough, it was worth mentioning that TPP‐LND@Lip had a more obvious inhibitory effect on tumor growth compared with the PD‐L1 antibody to some extent (Figure 8). Thus, TPP‐LND@Lip could better sensitize radiotherapy in vivo.

It is well known that the downregulation of PD‐L1 protein expression enhances the killing effect of T lymphocytes on tumors. Staining of LLC tumor sections showed that TPP‐LND@Lip significantly enhanced the infiltration and activation of CD3^+^, CD4^+^, and CD8^+^ T cells in the tumor microenvironment after radiotherapy (Figure 8F and Figures S32 and S33, Supporting Information). Besides, Ki67, TUNEL, and H&E staining were used to evaluate the antitumor effect of TPP‐LND@Lip in vivo. These findings were consistent with the concept that the proliferation inhibition rate and killing rate of TPP‐LND@Lip nanoparticles in LLC tumor cells after radiotherapy were significantly higher than those in other groups (Figure 8G). Generally speaking, TPP‐LND@Lip combined with radiotherapy could induce tumor cell death and slow tumor cell growth to the greatest extent and was highly likely to be a perfect substitute for PD‐L1 antibody.

### 2.9 Safety Evaluation of TPP‐LND@Lip

**Figure 9:**
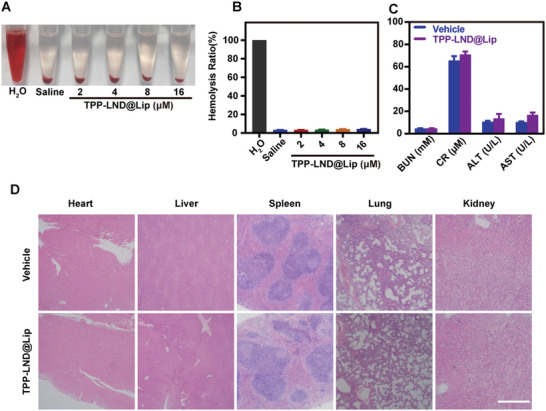
Safety evaluation of TPP‐LND@Lip in vivo. A) Photographs of hemolysis assay of different concentrations of TPP‐LND@Lip (double distilled water as the positive control, and saline as the negative control). B) Quantifications of hemolysis assay in (A). C) Assessment of liver and kidney function after treatment with TPP‐LND@Lip (10 mg kg^−1^) for 24 h (*n* = 3). D) Representative H&E images of the major organs (heart, liver, spleen, lung, and kidney) from normal mice treated with saline or TPP‐LND@Lip, scale bar = 100 µm. Data were presented as mean ± SD.

Finally, the safety of TPP‐LND@Lip nanoparticles in vivo was evaluated by hemolysis test, blood biochemical markers, and H&E staining. Hemolysis test analysis indicated that TPP‐LND@Lip did not cause hemolysis at various concentrations (**Figure** **9**A,B). Apart from this, after 24 h treatment with TPP‐LND@Lip, serum creatinine (CRE), blood urea nitrogen (BUN), aspartate aminotransferase (AST), and alanine aminotransferase (ALT) levels of mice were not significantly increased compared with the control group, which suggested that TPP‐LND@Lip had no significant effect on liver and kidney function (Figure 9C). Finally, H&E staining was performed on the heart, liver, spleen, lung, or kidney after killing the mice, and the assay confirmed that TPP‐LND@Lip had no obvious toxic or side effects on normal tissues (Figure 9D). On the whole, TPP‐LND@Lip had excellent safety in vivo and exceptional clinical application value in tumor treatment.

## 3 Discussion

At present, a great many methods was discovered for the treatment of lung cancer, among which radiation therapy and immunotherapy are two of the most important methods. However, the progress in curative effects of lung cancer therapy in recent decades is still limited. In this study, we newly found that LND could reduce the expression of PD‐L1 in the cytoplasm and cytomembrane of lung cancer cells by restraining mitochondrial OXPHOS (Figure 1). The underlying mechanism was the activation of the AMPK pathway. It is worth noting that TPP‐LND@Lip nanoparticles developed by us solved the problems of PD‐L1 up‐regulation and tumor hypoxia at the same time, and showed better radiosensitization compared with PD‐L1 antibodies. These findings opened new avenues for immunotherapy and radiotherapy for lung cancer.

Immunotherapy of lung cancer currently more commonly refers to immune checkpoint inhibitor therapy, such as Pabolizumab, Navulizumab, and Duvarizumab, which can be used alone or in combination with other therapies.^[^ ^24^ ^]^ However, it is recognized that radiation therapy, chemotherapy, photodynamic therapy, and other common cancer treatments are capable of leading to the increased expression of PD‐L1, thus weakening the activity of immune cells and the efficacy of immunotherapy.^[^ ^11^, ^25^ ^]^ In addition, new studies have shown that intracellular PD‐L1 severely limits the effectiveness of radiation or chemotherapy by increasing the resistance of cells to DNA damage.^[^ ^6a^ ^]^ Therefore, the traditional anti‐PD‐1/PD‐L1 monoclonal antibody only destroys the receptor‐ligand binding on the cell membrane surface but fails to affect the intracellular PD‐L1 mediated signaling pathway in cancer cells, resulting in poor sensitization radiotherapy effect in lung cancer treatment.

Currently, some small molecules that clear both tumor membranal and intracellular PD‐L1 may serve as more potent PD‐L1 inhibitors.^[^ ^8^, ^25^ ^]^ As proved before, the small‐molecule anti‐tumor drug LND plays an anticancer role mainly by affecting the energy metabolism of cells.^[^ ^26^ ^]^ Nevertheless, its regulation of PD‐L1 protein expression in lung cancer cells has not been thoroughly studied. Our experiment confirmed that LND could reduce the expression of PD‐L1 in and out of the lung tumor cells by affecting the mitochondrial respiratory chain (Figure 1). However, such an effect could only be achieved at a high dose of LND due to its strong hydrophobicity and weak targeting of tumor tissues, hence it had great toxic and side effects on normal tissues, which affected its wide clinical application. Previous studies have shown that mitochondria‐targeted LND could significantly reduce the dose required for LND to be effective.^[^ ^15^ ^]^ Therefore, for the sake of solving the above problems, TPP^+^ was chemically coupled with LND and further loaded with liposome to construct the TPP‐LND@Lip nanoplatform targeting tumor mitochondria (Figure 2). Not only could it affect mitochondrial energy biology to reverse the high expression of PD‐L1 by selectively targeting tumors, but also, possessed better effects when combined with radiotherapy compared with PD‐L1 antibody (Figures 6, 7, 8). In summary, TPP‐LND@Lip, constructed by a simple and economical method, solved the systemic adverse reactions and insufficient efficacy of traditional PD‐L1 antibodies and could be used as a new strategy for tumor‐targeted regulation of PD‐L1.

It is known that the hypoxic environment of tumors also reduces DNA damage and induces resistance of tumor cells to radiation or chemotherapy.^[^ ^27^ ^]^ The exciting news is that TPP‐LND@Lip could improve tumor oxygenation to reduce endogenous oxygen consumption by inducing mitochondria‐related metabolic intervention. Our study also confirmed that TPP‐LND@Lip solved the problems of PD‐L1 upregulation and tumor hypoxia, which had an excellent radiosensitization effect (Figures 4, 5, 6, 7, 8). Considering the fact that chemotherapy and photodynamic therapy are both oxygen or PD‐L1‐dependent tumor therapies, we firmly believe that TPP‐LND@Lip could also be applied to sensitize chemotherapy and photodynamic therapy. Additionally, we have demonstrated that TPP‐LND@Lip down‐regulated PD‐L1 expression via the AMPK pathway in MB49 cells (Figure S8, Supporting Information). However, whether TPP‐LND@Lip can promote radioimmunotherapy for more tumor types deserves further investigation. In a recent study of triple‐negative breast cancer, D‐mannose inhibited PD‐L1‐mediated DDR and sensitized tumor cells to ionizing radiation.^[^ ^6c^ ^]^ Radiotherapy up‐regulated the expression of PD‐L1 in nasopharyngeal carcinoma cells and PD‐1 in NK cells. Blocking the PD‐L1/PD‐1 checkpoint enhanced the killing effect of radiotherapy on nasopharyngeal carcinoma cells, even though this killing effect was achieved by enhancing the activity of NK cells.^[^ ^28^ ^]^ Theoretically, TPP‐LND@Lip is possible to play a similar role in other cancers treated by radiotherapy with high PD‐L1 expression. In a few words, our study revealed the link between mitochondrial metabolism and immune resistance, developed new functions of TPP‐LND@Lip in lung cancer radioimmunotherapy and provided new ideas for addressing the limitations of radiotherapy and PD‐L1 antibody therapy.

AMPK is a key molecule in the regulation of biological energy metabolism, which expresses in various metabolism‐related organs and serves as the nucleus for the study of diabetes mellitus and other metabolism‐related diseases.^[^ ^29^ ^]^ Of note, the AMPK activators metformin and 5‐aminimidazole‐4‐carboxyamide 1‐*β*‐d‐nuclear furanoside have been shown to inhibit tumor progression.^[^ ^30^ ^]^ AMPK also promotes autophagy by inhibiting the mTOR signaling pathway.^[^ ^15^, ^31^ ^]^ Therefore, the direct or indirect activators of AMPK not only improve metabolic diseases including diabetes, obesity, and cardiovascular diseases but also are expected to play an inestimable role in cancer radiotherapy by inhibiting the signal of promoting proliferation, regulating the process of cell cycle and participating in the regulation of autophagy.^[^ ^32^ ^]^ In this study, the AMPK indirect activator TPP‐LND@Lip was economical and simple to synthesize, safe and effective in vivo, and may also prevent or delay the occurrence or development of energy metabolic diseases to a large extent.

## 4 Conclusion

In this research, we demonstrated that the OXPHOS suppressor drug LND induced downregulation of PD‐L1 expression in lung tumor cells by affecting energy metabolism. To solve the limitation that LND was unable to be widely used in the clinic due to the high dose required, we chemically conjugated it with the mitochondrial targeting group TPP^+^ and transformed it into TPP‐LND@Lip nanoparticles for further functional exploration. Due to the targeting of the chemical group TPP^+^, TPP‐LND@Lip was selectively transferred into the tumor mitochondria, and its inhibitory effect on OXPHOS was about 50 times that of LND@Lip. Besides, in terms of PD‐L1 expression regulation, TPP‐LND@Lip could achieve a satisfactory effect at a relatively low dose (2 µm) compared with LND reducing PD‐L1 expression at a high dose (300 µm). Given that TPP‐LND@Lip also down‐regulated intracellular PD‐L1, it was safer, cheaper, and more effective than PD‐L1 antibodies to some extent, which was relatively expensive and acted only on the cell surface. In addition, TPP‐LND@Lip had the ability to reverse tumor hypoxia and induce more ROS production when combined with radiation therapy. Finally, TPP‐LND@Lip synergistically increased DNA damage and sensitized radiotherapy via PD‐L1 downregulation and hypoxia reversion by inhibiting mitochondrial OXPHOS, which then significantly inhibited the growth of lung cancer. In a word, the multifunctional nanoparticle TPP‐LND@Lip developed by us could sensitize the radioimmunotherapy of lung cancer through metabolic reprogramming, which may be used as a possible alternative of PD‐L1 antibody in the clinical treatment of tumors.

## 5 Experimental Section

### Materials

Butyltriphenylphosphonium bromide (TPP‐C4) was bought from Sigma‐Aldrich. 4‐bromobutan‐1‐amine hydrobromide, TPP, acetonitrile, dichloromethane, methanol (MeOH), LND, and 4‐(4,6‐Dimethoxy‐1,3,5‐triazin‐2‐yl)‐4‐methylmorpholinium chloride (DMTMM) were obtained from Aladdin Co., Ltd. (Shanghai, China). DCFH‐DA and JC‐1 assay kits were purchased from Beyotime Biotechnology (Shanghai, China). All reagents of the western blotting assay were acquired from Beyotime Biotechnology (Shanghai, China). Various detection kits and antibodies were obtained from various sources, which are listed in their respective method sections. All biochemical reagents were research‐grade and had not undergone further purification.

### Synthesis of TPP‐LND

Recently, it was revealed that AMPK activation via mitochondria OXPHOS depression was closely related to PD‐L1 downregulation.^[^ ^6c^ ^]^ It was known to all that LND was confirmed to inhibit OXPHOS by mitochondrial complex Ι and II suppression, the effective dose of which was relatively too high.^[^ ^14^, ^16^ ^]^ It is worth noting that the effective dose and side effects of LND can be significantly reduced by coupling LND with TPP^+^ to form a mitochondrial‐targeted delivery system.^[^ ^15^, ^17^ ^]^ In this study, TPP‐LND was synthesized by the following methods.

Synthesis of [Ph_3_PC_4_H_8_NH_2_]^+^Br^−^ (TPP‐C4): TPP‐C4 was obtained by the reaction between 2.32 g 4‐bromobutan‐1‐amine hydrobromide and 3.92 g TPP in 80 mL acetonitrile. Then, the solution was stirred and refluxed at 80 °C for 18 h. Following this, the reaction mixture was rotor‐evaporated, and the rest of the crystals were dissolved in a minimal amount of water. Thereafter, the water was rotor‐evaporated and the amine was extracted with MeOH. The final product was further purified by high‐performance preparation liquid (yield 42.3%, ≈95% pure).

Synthesis of TPP‐LND: TPP‐LND was obtained by the reaction between 826 mg TPP‐C4, 588 mg DMTMM, 202 mg trimethylamine, and 642 mg LND in 40 mL ethanol and 40 mL dichloromethane. Then, the solution was stirred and reacted at room temperature for 48 h. Following this, the reaction mixture was rotor‐evaporated and further purified by high‐performance preparation liquid (yield 56.3%, ≈95% pure).

Characterization data for TPP‐LND: ^1^H NMR (400 MHz, CDCl_3_) *δ* 8.33 (d, *J* = 8.1 Hz, 1H), 7.85 (dd, *J* = 12.6, 7.7 Hz, 5H), 7.69 (dd, *J* = 8.5, 6.5 Hz, 3H), 7.60 (td, *J* = 7.6, 3.2 Hz, 6H), 7.41 (t, *J* = 3.4 Hz, 3H), 7.31–7.23 (m, 1H), 7.12 (dd, *J* = 8.4, 2.1 Hz, 1H), 6.94 (d, *J* = 8.4 Hz, 1H), 5.67 (s, 2H), 4.25–3.88 (m, 2H), 3.64 (d, *J* = 6.0 Hz, 2H), 1.76 (q, *J* = 8.1, 7.7 Hz, 2H), 1.26 (s, 1H). ^13^C NMR (100 MHz, CDCl_3_) *δ* (ppm) = 163, 141, 138.3, 134.80 (d, *J* (C, P) = 2.8 Hz), 134.50, 133.80 (d, *J* (C, P) = 10 Hz), 133.2, 132.3, 130.5, 130.3 (d, *J* (C, P) = 12.6 Hz), 129.3, 127.8, 127.2, 123, 122.9, 122.7, 118.5 (d, *J* (C,P) = 85.8 Hz), 109.4, 50, 37.5, 29.8 (d, *J* (C, P) = 16.8 Hz), 19.7, 22 (d, *J* (C, P) = 50 Hz).

### Preparation of TPP‐LND@Lip

TPP‐LND@Lip was prepared by the thin film hydration method. First, 70 mg of HSPC, 30 mg of cholesterol, and 3 mg of TPP‐LND were dissolved in chloroform. The mixture was then evaporated on a rotary evaporator to form a lipid dry film in a round‐bottomed flask. Afterward, double steaming water (2 mL) was added, followed by ultrasonic treatment in the water bath for 10 min. The prepared liposomes were extruded 20 times with an extruder (LiposoFast, Avastin, Canada) through 200 nm polycarbonate membrane filters (Merck Millipore Ltd., USA). Then, the free drugs were isolated by Amicon Stirred Cells with a 30 kDa ultrafiltration membrane (Limin Industrial Co., Ltd., Shanghai, China). The final sample (≈10 mL) was stored at 4 °C or freeze‐dried. The final liposome concentration was about 500 µm. Blank Lip, TPP‐C4 liposome (TPP‐C4@Lip), lonidamine liposome (LND@Lip), liposomes containing Chlorin e6 (Ce6) and TPP‐LND (Ce6@TPP‐LND@Lip), and liposomes containing indocyanine green (ICG) and TPP‐LND (ICG@TPP‐LND@Lip) were prepared by a similar method. The dosage of the drugs in these liposomes was consistent with TPP‐LND@Lip.

### Characterization of These Nanoparticles

The morphology of liposomes was observed by transmission electron microscopy (TEM, FEI Talos F200S, Thermo Fisher Science, USA). The size distribution and Zeta potential of the liposomes were determined by Zetasizer (Zetasizer Nano ZS ZEN3600, Malvern, UK). The loading rate and encapsulation rate of TPP‐C4, LND, and TPP‐LND were determined by the high‐performance liquid chromatography (Agilent1260, USA) and UV–vis spectrometer (Lambda 25, PerkinElmer, USA), respectively.

### Cell Culture

Human lung A549 cancer cells and mouse LLC lung cancer cells were cultured in DMEM medium supplemented with 10% FBS and 1% penicillin/streptomycin, respectively, and in an incubator at 37 °C and 5% CO_2_. Mouse bladder cancer cell line MB49 was cultured in 1640 medium containing 10% FBS and 1% penicillin/streptomycin under the same cell culture environment.

### Western Blot Assay In Vitro

1 × 10^6^ cells were inoculated in 6 cm cell culture dishes for 24 h, and then treated with different concentrations of LND, TPP‐LND, TPP‐C4, or the same concentration of TPP‐C4@Lip, LND@Lip, TPP‐LND@Lip (TPP‐C4, LND, and TPP‐LND: 3 µm), and fresh culture medium for 24 h as control. The cells were then washed with PBS and lysed with cold RIPA buffer. To obtain protein cleavage products, cells were scraped, cleaved for 20 min, and then centrifuged at 4 °C at 12000 rpm for 20 min. Next, the cleavage products were detected using the BCA protein analysis kit and denatured in a 95 °C bath. The same amount of protein was separated by SDS‐PAGE and transferred to the PVDF membrane. The PVDF membrane was closed with 5% skim milk and incubated with rabbit anti‐mouse *β*‐actin antibody (1:5000, affinity), rabbit anti‐mouse AMPK antibody (1:1000, CST), rabbit anti‐mouse p‐AMPK antibody (1:1000, CST), and rabbit anti‐mouse CD274 antibody (1:1000, affinity), respectively, at 4 °C overnight. After 24 h, they were co‐incubated with secondary antibodies at room temperature for another 2 h. Finally, the fluorescence chemiluminescence gel imaging system (Peiqing Science and Technology) was used to obtain the image of protein bands.

To evaluate the influence of radiotherapy on the expression of PD‐L1 protein, 1 × 10^6^ cells were inoculated in 6 cm cell culture dishes and cultured for 24 h. The cells were irradiated with 0, 2, 4, 6, 8, and 10 Gy, respectively, after which the cells were continued to culture for 24 h. To further determine the effect of the drug combined with radiotherapy on PD‐L1 expression, the cells were treated with TPP‐LND@Lip (TPP‐LND: 3 µm) or fresh culture medium as the control for 24 h and then irradiated with 6 Gy (3 Gy min^−1^, 2 min) followed by removing different treatments, continuing to be cultured for 24 h. PD‐L1 and *β*‐actin protein bands were obtained by the same method.

### Detection of ADP/ATP Ratio

1 × 10^4^ LLC cells were inoculated in 24‐well plates overnight, treated with different concentrations of TPP‐LND or different treatments (TPP‐C4@Lip, LND@Lip, and TPP‐LND@Lip: 3 µm) for 12 h, collected and cleaved. Finally, the ADP and ATP levels of cells were determined by the ADP/ATP Lite Assay Kit.

### Immunofluorescence Analysis In Vitro

1.5 × 10^5^ cells were inoculated into Confocal Dish for 24 h, and then cultured with different concentrations of TPP‐LND or the same concentrations of TPP‐C4@Lip (TPP‐C4: 3 µm), LND@Lip (LND: 3 µm), and TPP‐LND@Lip (TPP‐LND: 3 µm) for 24 h, respectively, followed by PBS washing for twice. Cells were treated with rabbit anti‐mouse HIF‐1*α* antibody (1:250, Biolegend) or rabbit anti‐mouse anti‐CD274 antibody (1: 250, Biolegend) at 37 °C for 2 h, then fixed with 4% paraformaldehyde at room temperature for another 15 min and washed with PBS for three times, after which the cells were stained with DAPI for 10 min and washed with PBS for two times. Finally, the fluorescence was detected by confocal laser microscopy.

PD‐1 binding assay can evaluate the expression level of PD‐L1. 1.5 × 10^5^ LLC cells were grown in a confocal culture dish for 24 h and then inoculated with TPP‐C4@Lip (TPP‐C4: 3 µm), LND@Lip (LND: 3 µm), or TPP‐LND@Lip (TPP‐LND: 3 µm) for 24 h, the cells were fixed in 4% paraformaldehyde for 15 min and then incubated with recombinant mouse PD‐1 Fc protein (1:500, Biolegend) overnight. Subsequently, sheep anti‐human lgG (Dylight 488, Invitrogen) was used as a secondary antibody and incubated at room temperature for 2 h. Finally, DAPI was used to stain the nucleus, and CLSM was used for image acquisition.

Similarly, to further verify the effect of radiotherapy on PD‐L1 expression and PD‐1 binding ability, cells were inoculated into a confocal dish for 24 h and cultured with TPP‐LND@Lip (TPP‐LND: 3 µm) or fresh culture medium as the control for 24 h, followed by irradiated with 6 Gy (3 Gy min^−1^, 2 min), after which the drug was removed and the cells were cultured for 24 h sequentially, and then rabbit anti‐mouse anti‐CD274 antibody (1: 250, Biolegend), recombinant mouse PD‐1 Fc protein (1: 500, Biolegend). Finally, intracellular fluorescence was detected by confocal laser microscopy.

DNA damage was determined by the amount of a DNA damage marker *γ*‐H_2_AX (i.e., phosphorylated H_2_AX). Cells were treated with TPP‐C4@Lip (TPP‐C4: 3 µm), LND@Lip (LND: 3 µm), or TPP‐LND@Lip (TPP‐LND: 3 µm) and fresh culture medium for 24 h, and then irradiated with 6 Gy (3 Gy min^−1^, 2 min). The cells were washed twice with PBS at 0, 4, and 24 h after irradiation and fixed with 4% paraformaldehyde at room temperature for another 15 min. In the next place, the cells were permeated in 0.5%Triton X‐100 for 10 min and then blocked at room temperature with a blocking solution containing bovine serum albumin for 1 h, the next, incubated with primary antibody *γ*‐H_2_AX (1:200, affinity) at 4 °C overnight. After that, Alexa Fluor488 labeled goat anti‐rabbit (H+L) secondary antibodies were incubated at room temperature for 1 h. Finally, the fluorescence was observed by confocal laser microscope after nuclear staining with DAPI.

### Assay of Mitochondrial Complexes Activity

LND and TPP‐LND were diluted into different concentrations and the activity of the mitochondrial Complex was determined by using the MitoCheck Complex Ι activity analysis kit and the MitoCheck Complex II Activity Analysis kit (Cayman, USA).

### Detection of Mitochondrial Membrane Potential

2 × 10^5^ LLC cells were inoculated in confocal culture dishes and cultured overnight. Second, the cells were incubated with TPP‐C4@Lip (TPP‐C4: 3 µm), LND@Lip (LND: 3 µm), or TPP‐LND@Lip (TPP‐LND: 3 µm) for 8 h. The blank control group was the negative control group, and CCCP was the positive control group for 20 min. Then JC‐1 probe was added into the cell culture medium for further treatment for 20 min. Finally, the MMP was measured by laser confocal microscopy.

### Cytotoxicity and Cell Clone Assay In Vitro

Cytotoxicity tests were performed on different cancer cell lines (LLC, A549, and MB49). In general, 8 × 10^3^ tumor cells were cultured in each well of 96‐well plates, and the culture time was 24 h. Subsequently, the cells were incubated with a culture medium containing different concentrations of LND@Lip, TPP‐C4@Lip, and TPP‐LND@Lip. After 24 h, the survival rate of the cells was detected by CCK‐8 assay, and half inhibitory concentration (IC50) was calculated.

For the sake of studying the promoting effect of TPP‐LND@Lip on radiotherapy cytotoxicity, LLC cells were inoculated into 96‐well plates with 5 × 10^3^ cells per well. After adherence, the cells were covered with paraffin wax for 12 h to provide a hypoxic environment. After incubation with different concentrations of TPP‐LND@Lip, LND@Lip, or TPP‐C4@Lip for 24 h, the cells were irradiated with 6 Gy (3 Gy min^−1^, 2 min), after which different treatments were removed, and the cells were cultured for 24 h unceasingly. CCK‐8 test solution was added to each well for 2 h afterward. In the end, the absorbance of UV light was measured at 450 nm by a microplate instrument.

To observe the effect of TPP‐LND@Lip on tumor inhibition over a longer period of time, the cell clonal formation experiment was also conducted. LLC cells of all groups were inoculated into 6‐well plates after being treated with TPP‐LND@Lip (TPP‐LND: 2 µm), radiation (6 Gy), and TPP‐LND@Lip (TPP‐LND: 2 µm) combined with radiation (6 Gy), respectively. After 12 days of culture, macroscopic cell colonies were formed and observed with crystal violet staining.

The cytotoxicity of TPP‐LND@Lip was verified by live/dead cell staining. After overnight inoculation in confocal culture dishes, cells were treated with TPP‐C4@Lip (TPP‐C4: 3 µm), LND@Lip (LND: 3 µm), or TPP‐LND@Lip (TPP‐LND: 3 µm), respectively. 24 h later, the cells were washed with PBS and stained with Calcein‐AM and PI for 30 min. Finally, the fluorescence of the cells was observed by a confocal laser microscope, and the living and dead cells were evaluated.

### T Cell‐Mediated Tumor Cell Killing Assay

The ethical approval was approved by the Medical Ethics Committee of the Second Affiliated Hospital of Wenzhou Medical University (No. 2022‐K‐138‐02). Human peripheral blood mononuclear cells (PBMC) were isolated from the blood of healthy donors. T cells were activated with immunobinding human CD3/ CD28 /CD2 T cell activator (25 µL mL^−1^, stem cells) for the first 3 days and cultured in amplification solution with IL‐2 (10 ng mL^−1^, PeproTech) was added for 7 days. Then A549 cells were treated with TPP‐LND@Lip (TPP‐LND: 3 µm) or fresh culture medium as the control for 24 h and stuck on the plate overnight. After that, the activated T cells and tumor cells in a 10:1 ratio were cultured in DMEM medium containing 10% FBS and 1% penicillin/streptomycin for 24 h, then T cells and cell fragments were washed with PBS, and live tumor cells were photographed by crystal violet staining.

### Detection of Reactive Oxygen Species

ROS probe DCFH‐DA (2′, 7′‐dichlorodihydrofluorescein diacetate) was used to detect the change in intracellular ROS concentration after irradiation. LLC cells were inoculated in confocal culture dishes and cultured in DMEM medium with or without TPP‐LND@Lip (TPP‐LND: 3 µm) for 24 h, and then replaced the drug with fresh cell culture medium containing 10 µ DCFH‐DA. After 20 min, the cells were irradiated with radiation (3 Gy min^−1^) for 2 min, and then the image was acquired by a laser confocal microscope.

### Animal Model

The ethical approval was approved by the Laboratory Animal Ethics Committee of Wenzhou Medical University (No. XM592021‐0214). To construct a mouse subcutaneous tumor model, 5 × 10^5^ LLC cells were suspended in 100 µL PBS and injected subcutaneously in the right groin of each C57BL/6 mouse (18‐20 g). As for the orthotopic lung cancer model, 5 × 10^5^ LLC cells were injected intravenously into a C57BL/6 mouse (18–20 g) via the tail vein. All animals were kept in an SPF environment and had free access to water and food.

### Imaging In Vivo

When the tumor volume reached 200 mm^3^, LLC tumor‐bearing mice were randomly divided into two groups and given Ce6 (2 mg kg^−1^) or Ce6@TPP‐LND@Lip (Ce6: 2 mg kg^−1^) intravenously, respectively. The mice were then anesthetized by intraperitoneal injection of 2% pentobarbital and imaged by in vivo fluorescent imaging system (IVIS Lumina XRMS Series III) at different time points (4, 12, 24, 36, or 48 h) after the injection. Tumor and normal tissues (heart, liver, spleen, lung, and kidney) were collected 48 h later for fluorescence imaging.

### Effect of Antitumor Therapy In Vivo

When the tumor volume of LLC reached about ≈100–120 mm^3^, mice were randomly divided into four groups: control group, TPP‐C4@Lip group, LND@Lip group, and TPP‐LND@Lip group, which were injected with normal saline, TPP‐C4@Lip (TPP‐C4: 7 mg kg^−1^), LND@Lip (LND: 7 mg kg^−1^), or TPP‐LND@Lip (TPP‐LND: 7 mg kg^−1^) through the tail vein. The tumor volume was then measured with a caliper, calculated as width^2^ × length × 0.5, and weight was also recorded. On the 14th day, the tumors were isolated and weighed.

In addition, in order to compare the anti‐tumor effects of anti‐PD‐L1 monoclonal antibody and TPP‐LND@Lip combined with radiotherapy, LLC tumor‐bearing mice were randomly divided into six groups: Saline, TPP‐LND@Lip (TPP‐LND: 7 mg kg^−1^), anti‐PD‐L1 monoclonal antibody (7 mg kg^−1^), radiotherapy (2.5 Gy min^−1^, irradiated for 2 min), anti‐PD‐L1 monoclonal antibody (7 mg kg^−1^) + radiation (2.5 Gy min^−1^, irradiation for 2 min), and TPP‐LND@Lip (TPP‐LND: 7 mg kg^−1^) + radiation (2.5 Gy min^−1^, irradiation for 2 min). X‐ray radiotherapy was performed 24 h after intravenous injection in different groups, and intravenous administration was performed on days 0, 4, and 7, respectively. Besides, tumor volume was measured with a caliper every 2 days, and weight was recorded continuously for 14 days. At last, the mice were killed to collect the tumors, which were then weighed and photographed.

When the tumor formed successfully in the lungs, the LLC tumor‐bearing mice were randomly divided into six groups: Saline, TPP‐LND@Lip (TPP‐LND: 7 mg kg^−1^), anti‐PD‐L1 monoclonal antibody (7 mg kg^−1^), radiotherapy (2.5 Gy min^−1^, irradiation for 2 min), anti‐PD‐L1 monoclonal antibody (7 mg kg^−1^) + radiation (2.5 Gy min^−1^, irradiation for 2 min), and TPP‐LND@Lip (TPP‐LND: 7 mg kg^−1^) + radiation (2.5 Gy min^−1^, irradiation for 2 min). X‐ray radiotherapy was performed 24 h after intravenous injection and intravenous administration was performed on days 0, 7, and 14, respectively. Bodyweight was recorded every 2 days. At the end of treatment, the mice were killed to collect the lungs, which were then weighed and photographed.

### Western Blot Assay and Immunofluorescence Analysis In Vivo

Tumor‐bearing mice were divided into four groups: Control group, TPP‐C4@Lip group, LND@Lip group, and TPP‐LND@Lip group. Saline, TPP‐C4@Lip (TPP‐C4: 7 mg kg^−1^), LND@Lip (LND: 7 mg kg^−1^), and TPP‐LND@Lip (TPP‐LND: 7 mg kg^−1^) were given to mice for 24 h, respectively. To analyze the expression of PD‐L1 in vivo, the tumor was ground in liquid nitrogen and lysed in cold RIPA for 20 min. The cleavage products were then tested with a BCA protein analysis kit and denatured in a 95 °C water bath. The same amount of protein was separated by SDS‐PAGE and transferred to the PVDF membrane afterward. Then, the PVDF membrane was closed with 5% skim milk and incubated with the primary rabbit anti‐mouse *β*‐actin antibody (1:5000, affinity) or rabbit anti‐mouse CD274 antibody (1:1000, affinity) overnight at 4 °C. 24 h later, they were co‐incubated with the secondary antibody at room temperature for 1 h. In the end, the fluorescence chemiluminescence gel imaging system (Peiqing Science and Technology) was used to obtain the image of protein bands. Further, to verify that TPP‐LND@Lip could reverse the upregulation of PD‐L1 after radiotherapy, LLC tumor‐bearing mice were divided into four groups: control group, radiotherapy group (2.5 Gy min^−1^, irradiation for 2 min), TPP‐LND@Lip group (TPP‐LND: 7 mg kg^−1^), and TPP‐LND@Lip (TPP‐LND: 7 mg kg^−1^) plus radiotherapy (2.5 Gy min^−1^, 2 min) group, radiotherapy was performed 24 h after administration. Similarly, PD‐L1 and *β*‐actin protein bands were obtained by the above method.

Two indicators, HIF‐1*α*, and pimonidazole were used to investigate the ability of TPP‐LND@Lip to reverse hypoxia in vivo. LLC tumor‐bearing mice (≈200 mm^3^) were randomly divided into four groups (three mice per group) and given normal saline, TPP‐C4@Lip (TPP‐C4: 7 mg kg^−1^), LND@Lip (LND: 7 mg kg^−1^), and TPP‐LND@Lip (TPP‐LND: 7 mg kg^−1^), which were sacrificed 24 h later. After that, tumor tissues were collected, embedded in OCT, and cut into sections with a thickness of 6 µm. The slices were then fixed in cold acetone for 10 min and punched with 0.3% Triton X‐100 for 30 min. Next, they were sealed with 3% bovine serum albumin at room temperature for 1 h and incubated with FITC labeled anti‐mouse HIF‐1*α* (1:250, Biolegend) overnight. Finally, DAPI staining was performed and laser confocal microscopy was used for fluorescence imaging. For the assessment of hypoxia based on pimonidazole, tumor‐bearing mice (≈200 mm^3^) were randomly divided into four groups, which were given Saline, TPP‐C4@Lip (TPP‐C4: 7 mg kg^−1^), LND@Lip (LND: 7 mg kg^−1^), or TPP‐LND@Lip (TPP‐LND: 7 mg kg^−1^), and pimonidazole hydrochloride (60 mg kg^−1^, Hypoxy Probe Inc., USA) was administered intravenously 24 h later. After another 2 h, all the mice were killed and tumors were made into sections, which were incubated with FITC‐labeled pimonidazole antibody (1:200) overnight at 4 °C. Finally, DAPI was used to stain the nucleus for 10 min, and the fluorescence images were observed by laser confocal microscope.

To demonstrate the tumor targeting capacity of TPP‐LND@Lip, Ce6, ICG, Ce6@TPP‐LND@Lip (2 mg kg^−1^ Ce6), or ICG@TPP‐LND@Lip (2 mg kg^−1^ ICG) were administered intravenously in LLC tumor‐bearing mice (*n* = 3). At 12, 24, and 48 h, tumors were collected from mice, embedded with OCT, and rapidly frozen with liquid nitrogen. After that, a freezing microtome was used to cut the tumor. The tumor sections were then immobilized in cold acetone and the nuclei were stained with DAPI. Finally, the fluorescence intensity of Ce6 and ICG in tumor tissues was observed by CLSM.

The effects of different therapeutic methods on the T‐cell infiltration, apoptosis, and proliferation of tumor cells were analyzed in this study. First, frozen tumor sections of 6 µm thickness were soaked in acetone for 15 min, and then gently washed with phosphate buffer. Next, the sections were sealed with 5% BSA and incubated with FITC‐labeled anti‐mouse CD274 (1:250, Biolegend), FITC‐labeled anti‐mouse CD3 (1:250, Biolegend), FITC‐labeled anti‐mouse CD4 (1: 250, Biolegend), FITC‐labeled anti‐mouse CD8 (1:250, Biolegend), FITC‐labeled anti‐mouse Granzyme B (1:250, Biolegend), FITC‐labeled anti‐mouse perforin (1:250, Invitrogen), and FITC‐labeled anti‐mouse IFN‐*γ* (1:250, Invitrogen) antibodies for 4 h. Then, the sections were washed with PBS and stained with DAPI. Finally, CLSM was used for image acquisition. At the end of the radiotherapy study, the mice were killed for tumor collection, and hematoxylin‐eosin (H&E) staining was used for histopathological analysis. What is more, tumor tissue sections were stained using the one‐step TUNEL kit (Beyotime, Shanghai, China), and tumor tissue apoptosis rates were detected a by confocal laser microscope (Nikon, Japan). To evaluate Ki67‐based cell proliferation, the samples were immobilized and inoculated with rabbit anti‐mouse Ki67 antibody (1:200, Affinity, USA) overnight and then incubated for another 1 h with Alexa Fluor 594 labeled goat anti‐rabbit antibody IgG (H+L) (Thermo Scientific, USA) at room temperature. At last, the fluorescence images were observed by laser confocal microscopy.

### Biosafety Evaluation

C57BL/6 mice were injected intravenously with TPP‐LND@Lip (TPP‐LND: 10 mg kg^−1^) or equal‐volume normal saline. Blood was taken from the eyeballs of mice 24 h later and centrifuged at 1200 rpm for 5 min at 4 °C. Finally, serum CRE, BUN, ALT, and AST levels were measured with the kit provided by Jiancheng Bioengineering Institute (Nanjing, China) to evaluate hepatic and renal function.

Furthermore, the hemolysis activity of TPP‐LND@Lip was determined by red blood cells (RBC). RBCs (40 µL) were added to TPP‐LND@Lip (100 µL) with different concentrations (TPP‐LND: 0, 2, 4, 8, and 16 µm) and double‐distilled water was used as the positive control. After standing at 37 °C for 2 h, the supernatant was obtained by centrifugation. Then the absorbance of the supernatant treated with different conditions at 540 nm was measured with a plate reader.

Last but not least, after 24 h treatment with TPP‐LND@Lip (TPP‐LND: 10 mg kg^−1^), major organs (heart, liver, spleen, lung, and kidney) were fixed with 4% paraformaldehyde, the tissue damage of which was observed by H&E staining.

### Statistical Analysis

Data were shown as the mean ± SD of three independent experiments. Shapiro–Wilk test was used to test for normal distribution and Levene's test was used to test for homogeneity of variance. Student's *t*‐test was used for pairwise comparisons. One way ANOVA test with Tukey's post‐hoc test was applied for multiple comparisons. * *p* < 0.05 was considered statistically significant; ** *p* < 0.01 and *** *p* < 0.001 were extremely significant; NSD, no significant difference.

## Conflict of Interest

The authors declare no conflict of interest.

## Author Contributions

C.X., Z.Z., and J.S. conceived the project. S.W., Z.Z., M.D., X.Z., S.R., Y.Z., and C.C. performed the experiments and analyzed the results. C.X. and J.S. provided useful suggestions for this work. S.W., M.Z., W.X., and L.H. wrote the manuscript. S.W., Z.Z., J.S., and C.X. discussed the results and reviewed the manuscript. Thanks to Prof. Fengming Kong for guidance and helpful suggestions.

## Supporting information

Supporting Information

## Data Availability

The data that support the findings of this study are available in the supplementary material of this article.
