# Correction to “Metabolic Intervention Liposome Boosted Lung Cancer Radio‐Immunotherapy via Hypoxia Amelioration and PD‐L1 Restraint”

**DOI:** 10.1002/advs.202509211

**Published:** 2025-06-23

Saijun Wang, Zaigang Zhou, Rui Hu, Mingyue Dong, Xiaobo Zhou, Siyan Ren, Yi Zhang, Chengxun Chen, Ruoyuan Huang, Man Zhu, Wanying Xie, Ling Han, Jianliang Shen, Congying Xie

Adv Sci (Weinh). 2023 Jun 23;10(18): 2207608.

https://doi.org/10.1002/advs.202207608


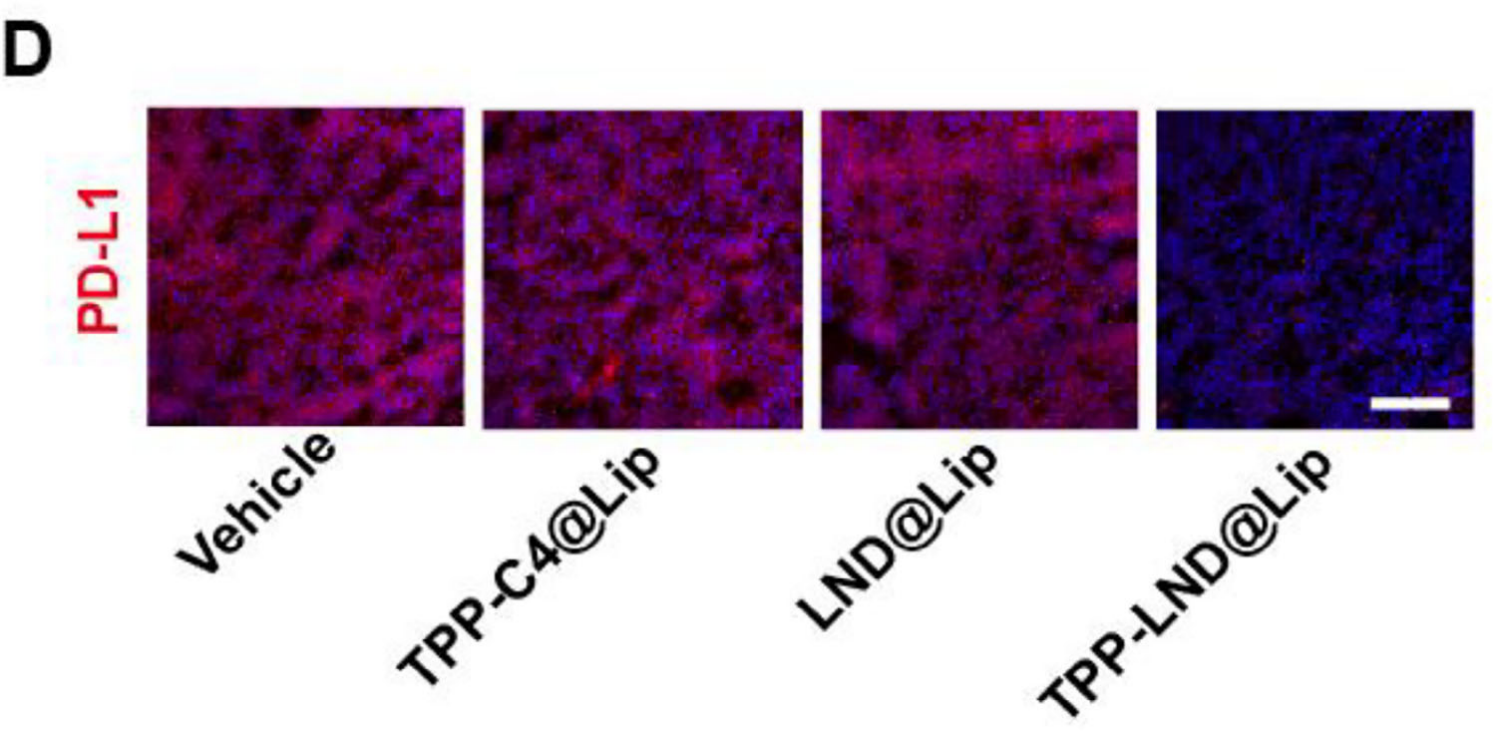


In Figure 5D, the figure shown above was incorrect, as the “TPP‐LND@Lip” image was mistakenly chosen from the group of “TPP‐C4@Lip” during images processing and assembly. We kindly ask for Figure 5D to be corrected as follows:

Corrected Figure 5D


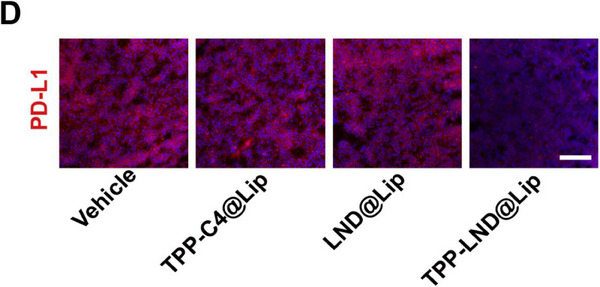



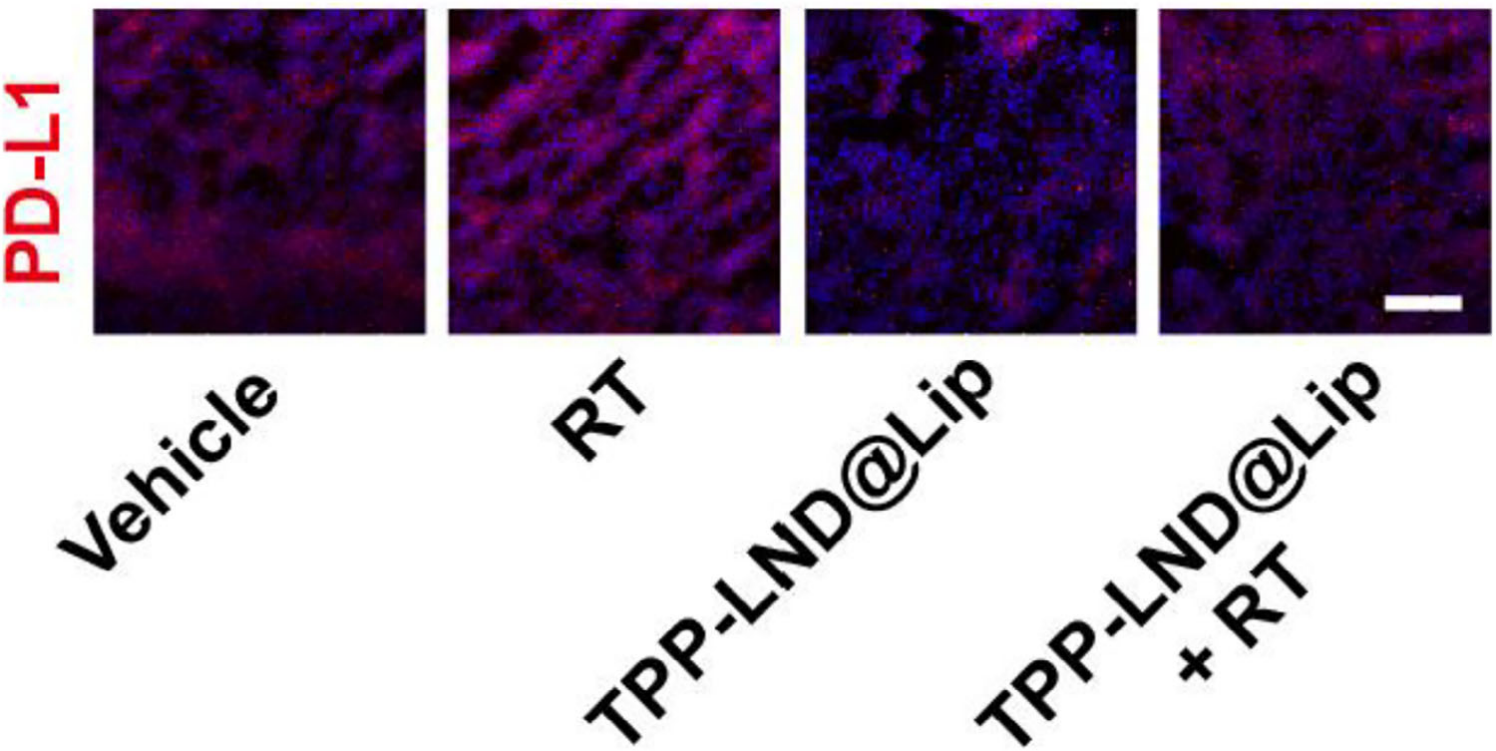


In Figure 7L, the figure shown above was incorrect, as the “Vehicle” and “TPP‐LND@Lip + RT” images were mistakenly chosen from the groups of “RT” and “LND@Lip” respectively during images processing and assembly. The corrected Figure 7L is shown below.

Corrected Figure 7L


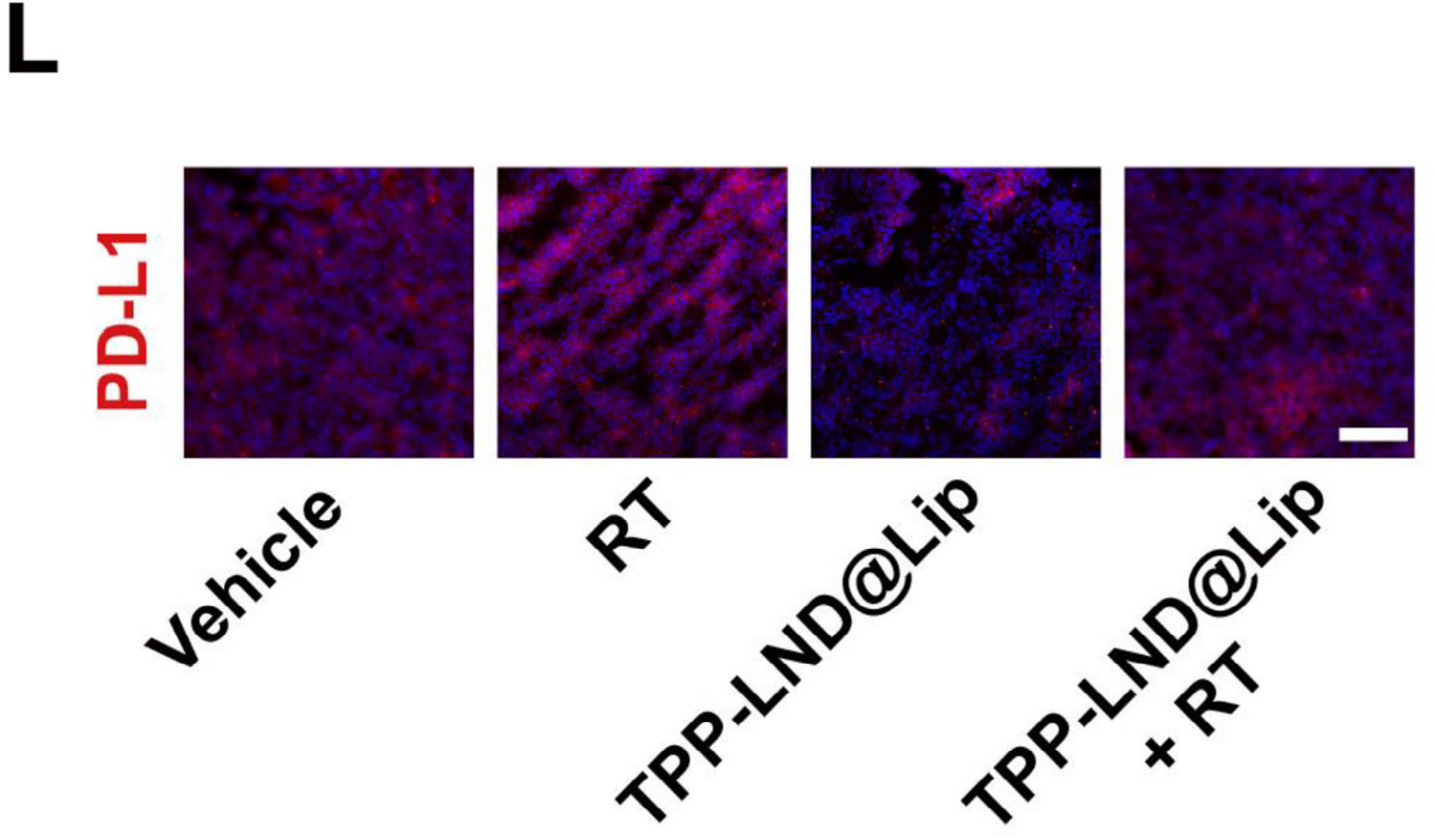



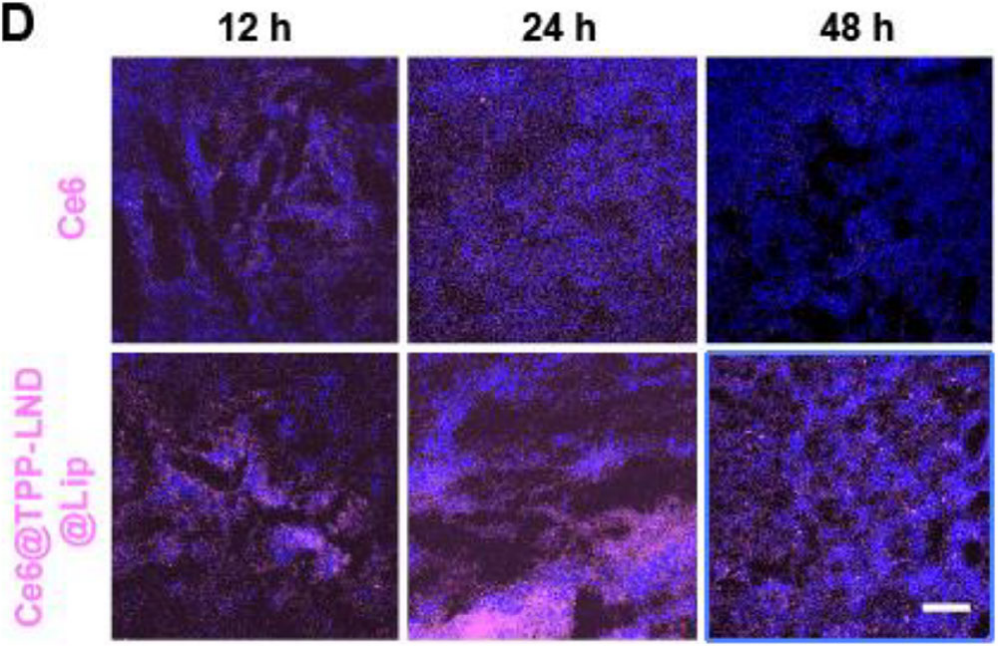


In Figure S21D (Supporting Information), the figure shown above was incorrect, as the “Ce6” image of the 12 h group was mistakenly represented as images of 48 h and Ce6@TPP‐LND@Lip. We kindly request for Figure S21D (Supporting Information) to be corrected as follows:

Corrected Figure S21D (Supporting Information)


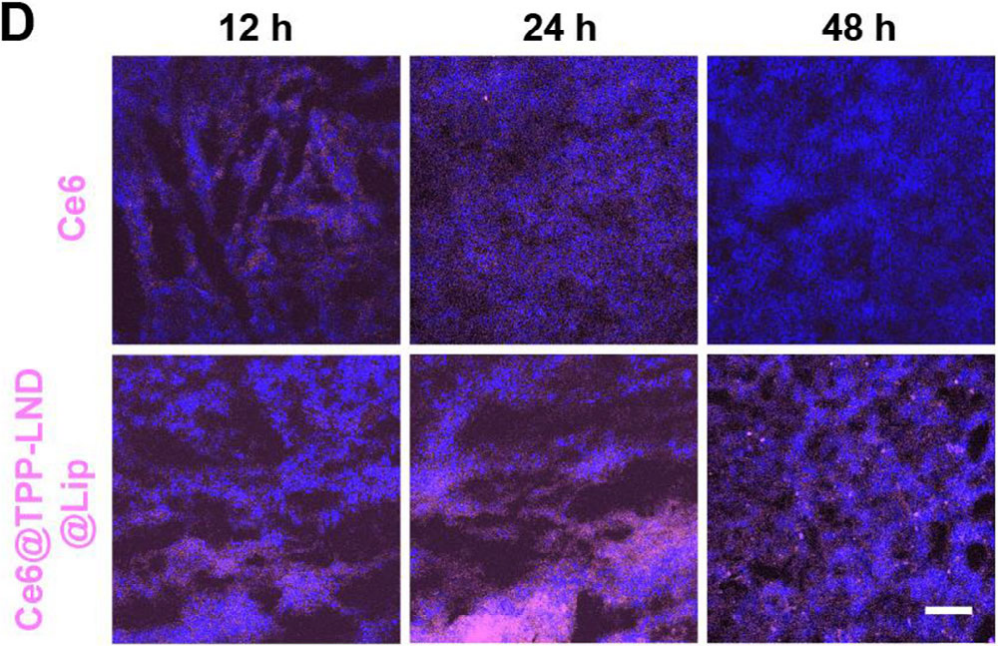



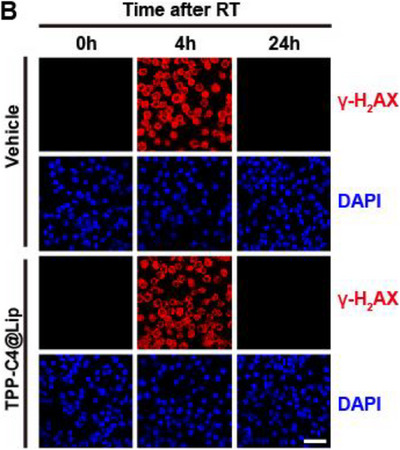


In Figure S24B (Supporting Information), the figure shown above was incorrect, as the image of “TPP‐C4@Lip 24h DAPI” group was erroneously duplicated for the “Vehicle 24h DAPI” group during the layout of the images. The corrected Figure S24B (Supporting Information) is presented below.

Corrected Figure S24B (Supporting Information)


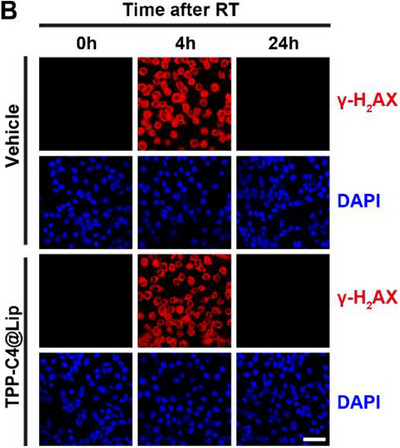


These corrections do not affect the overall findings and conclusions of the paper. We sincerely apologize for these errors.

