# Supplementary material for: Metabolic Intervention Liposome Boosted Lung Cancer Radio‐Immunotherapy via Hypoxia Amelioration and PD‐L1 Restraint
Source: Adv Sci (Weinh). 2023 Apr 24;10(18):2207608. doi: 10.1002/advs.202207608 (PMC10288235; doi:10.1002/advs.202207608)
Supplement: Supplementary file 1 — Supporting Information [file ADVS-10-2207608-s001.pdf]

Supporting Information

Metabolic Intervention Liposome Boosted Lung Cancer Radio-immunotherapy via Hypoxia Amelioration and PD-L1 Restraint

Saijun Wang [a], Zaigang Zhou [b,c,d,*], Rui Hu [a], Mingyue Dong [a], Xiaobo Zhou [a], Siyan Ren [a], Yi Zhang [a], Chengxun Chen [a], Ruoyuan Huang [a], Man Zhu [a], Wanying Xie [a], Ling Han [a], Jianliang Shen [b,c,d,e,*], Congying Xie [a,c,d,f,*]

[a] Medical and Radiation Oncology, Department of the Second Affiliated Hospital of Wenzhou Medical University, Wenzhou, 325000, China.

[b] State Key Laboratory of Ophthalmology, Optometry and Vision Science, School of Ophthalmology and Optometry, School of Biomedical Engineering, Wenzhou Medical University, Wenzhou 325027, China.

[c] Zhejiang Engineering Research Center for Innovation and Application of Intelligent Radiotherapy Technology, The Second Affiliated Hospital of Wenzhou Medical University, Wenzhou, Zhejiang 325000, China.

[d] Wenzhou Key Laboratory of Basic Science and Translational Research of Radiation Oncology, Wenzhou, Zhejiang 325000, China.

[e] Wenzhou Institute, University of Chinese Academy of Sciences, Wenzhou, Zhejiang 325000, China.

[f] Zhejiang-Hong Kong Precision Theranostics of Thoracic Tumors Joint Laboratory, The Second Affiliated Hospital of Wenzhou Medical University, Wenzhou, Zhejiang 325000, China.

*** Corresponding to:**

wzxiecongying@wmu.edu.cn (C. Xie), shenjl@wiucas.ac.cn (J. Shen), zhouzaigang1990@wmu.edu.cn (Z. Zhou).

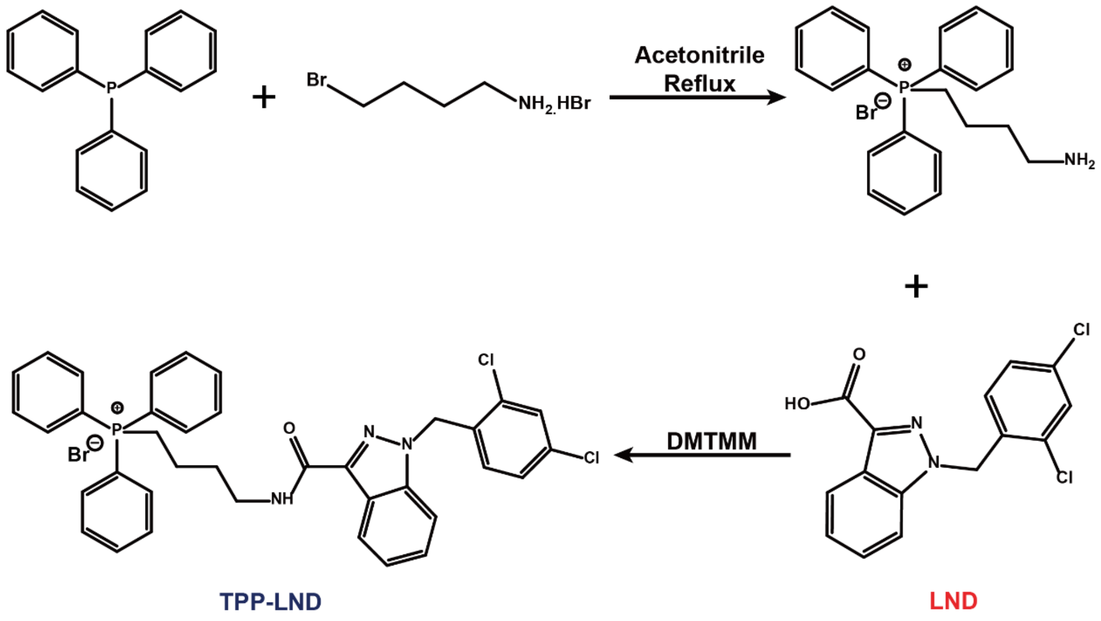

Figure S1. Synthetic route of TPP-LND.

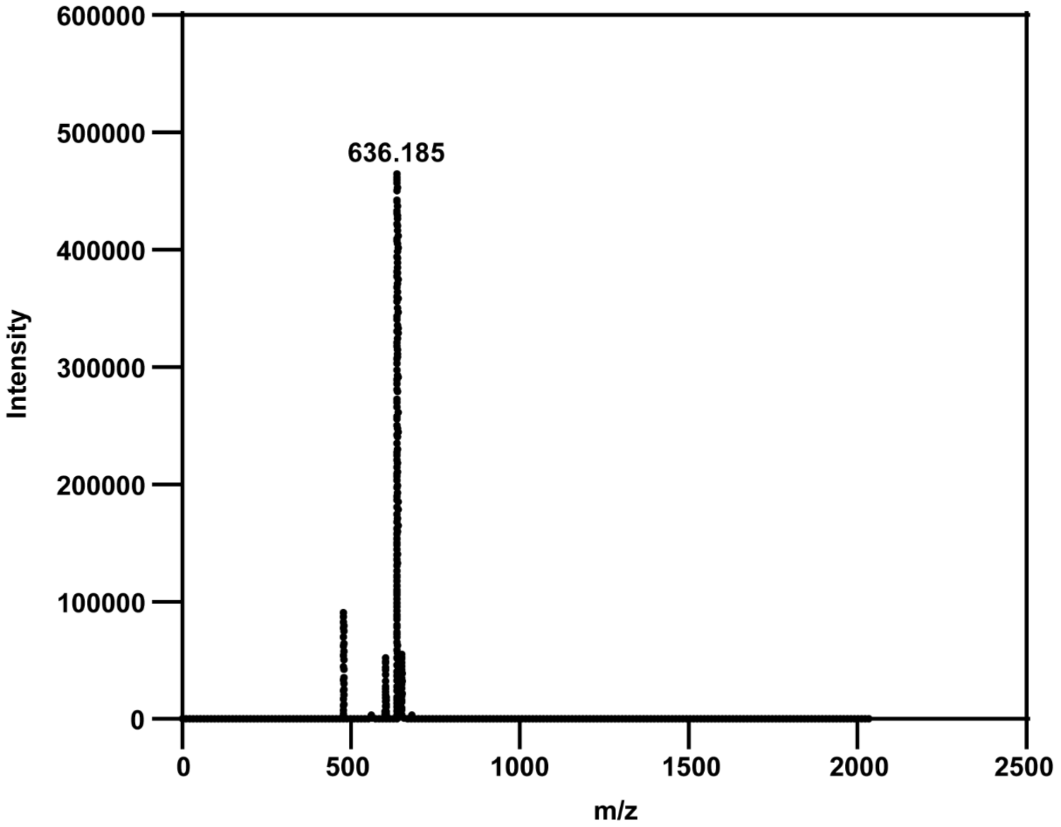

Figure S2. High-Resolution Mass Spectrometer (HR-MS) of TPP-LND.

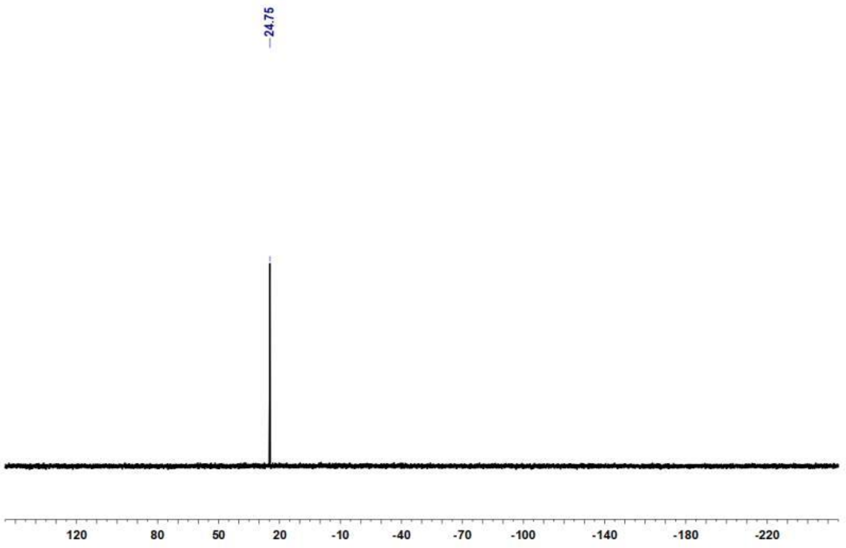

Figure S3. Phosphorus Magnetic Resonance Spectroscopy of TPP-LND.

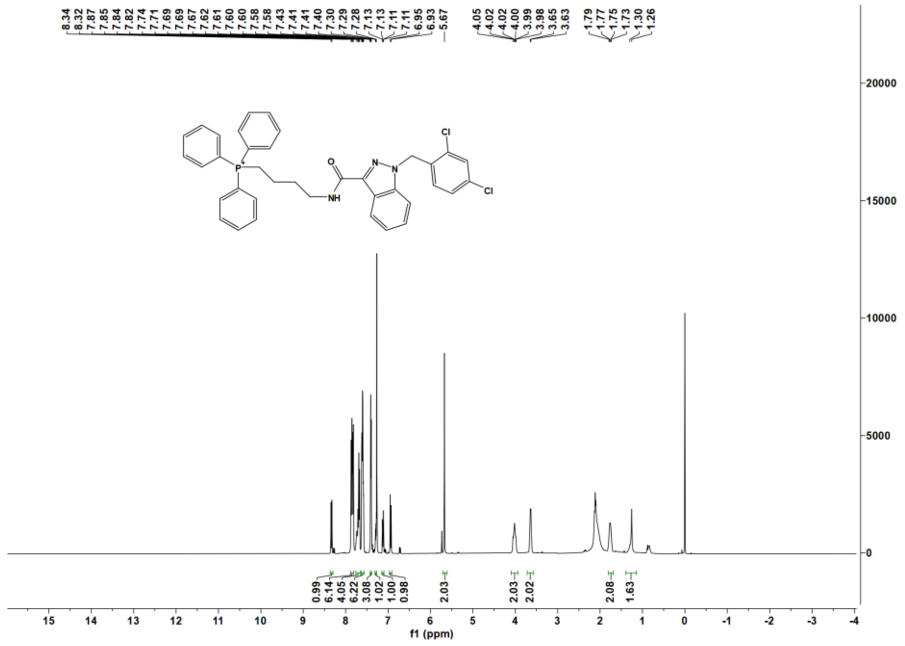

Figure S4. The ^{1}H-NMR spectrum of TPP-LND.

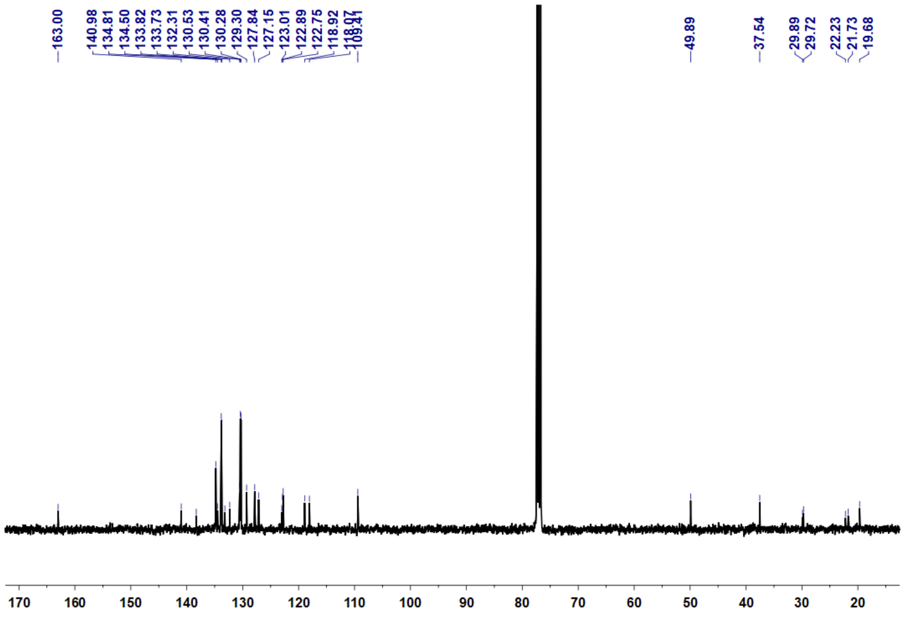

Figure S5. The ^{13}C-NMR spectrum of TPP-LND.

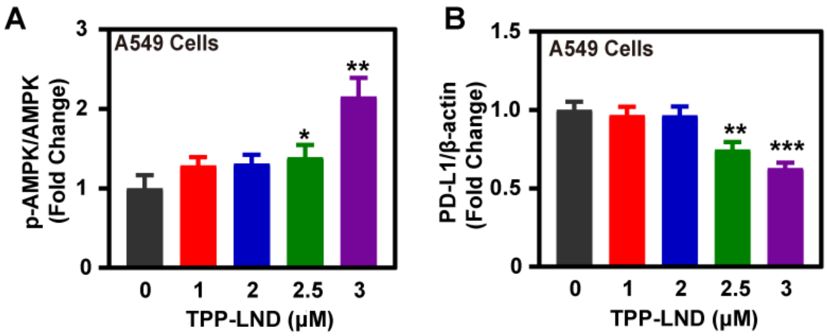

Figure S6. Determination of the p-AMPK and PD-L1 protein expression levels in A549 cells after treatment with different dosages of TPP-LND for 24 h. Data were presented as mean ± SD (n = 3). Statistical analysis was performed via the two-tail Student's *t*-test. * $p < 0.05$, ** $p < 0.01$, and *** $p < 0.001$.

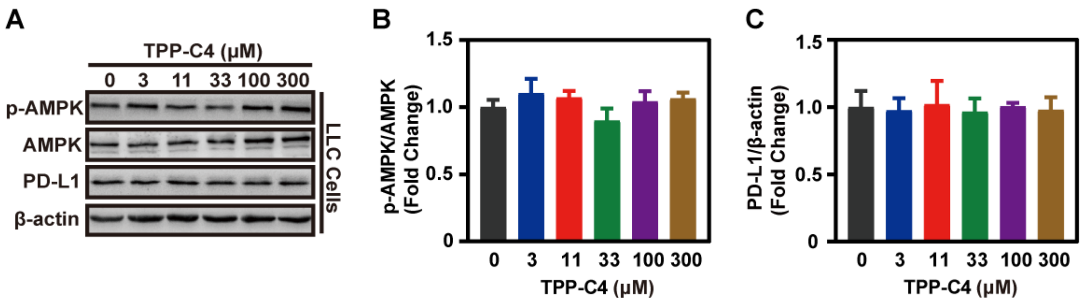

Figure S7. Expression of PD-L1, p-AMPK, and AMPK protein in LLC cells by Western Blot assay after treatment with different doses of TPP-C4 for 24 h and further quantification by ImageJ. Data were presented as mean ± SD (n = 3).

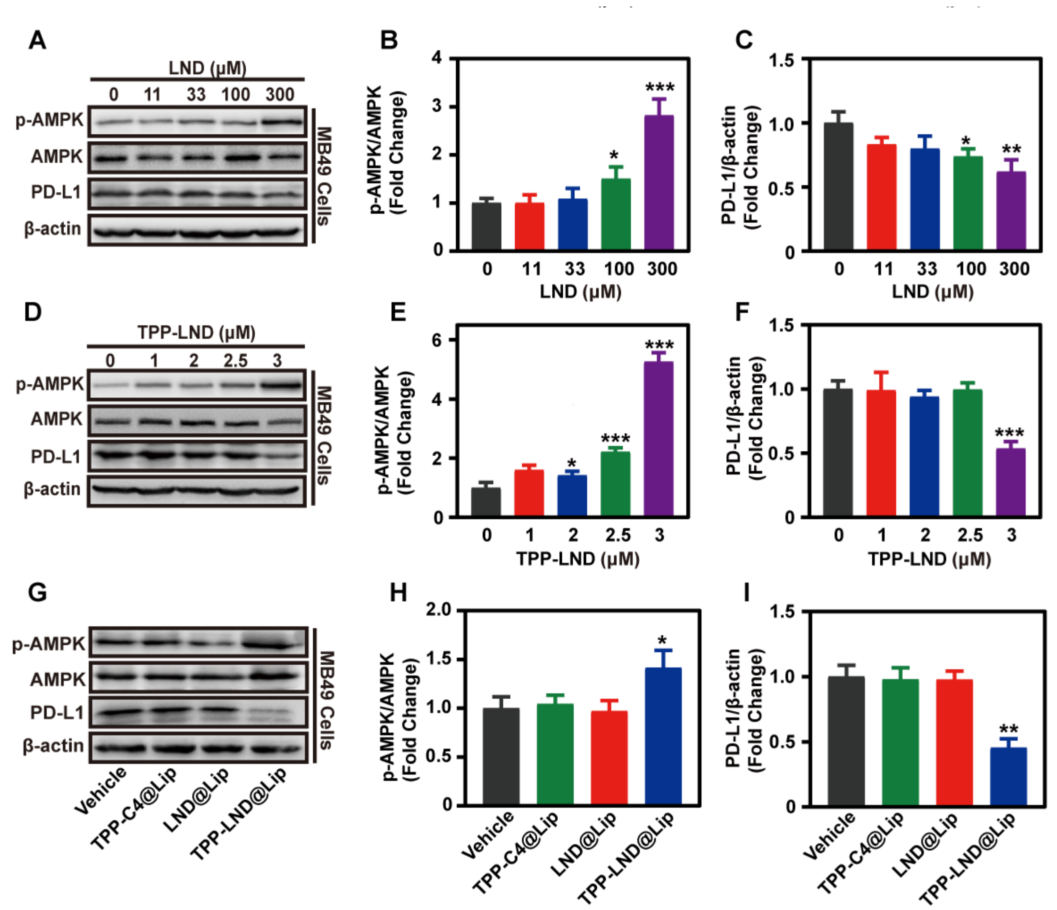

Figure S8. Effects of LND, TPP-LND, or TPP-LND@Lip on PD-L1, AMPK, and p-AMPK protein expression in MB49 cells by Western Blot assay after various treatments for 24 h and further quantification by ImageJ. Data were presented as mean ± SD (n = 3). Statistical analysis was performed via the two-tail Student's *t*-test. * $p < 0.05$, ** $p < 0.01$, and *** $p < 0.001$.

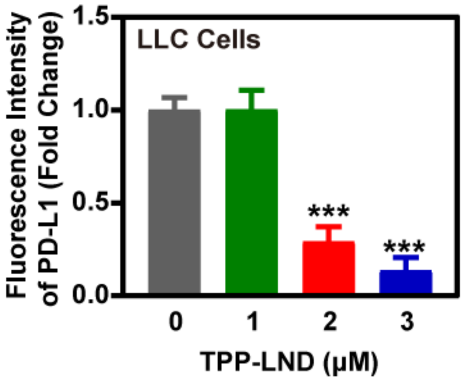

Figure S9. The normalized fluorescence intensity of PD-L1 protein quantified by Image J from Fig. 1K. Data were presented as mean ± SD (n = 3). Statistical analysis was performed via the two-tail Student's *t*-test. * $p < 0.05$, ** $p < 0.01$, and *** $p < 0.001$.

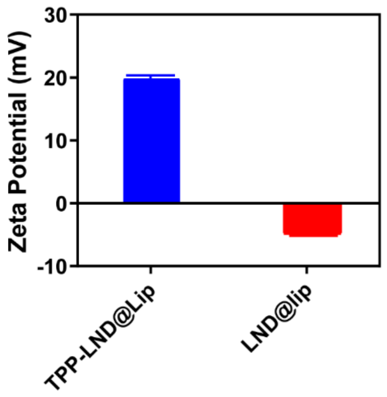

Figure S10. Zeta potential of TPP-LND@Lip and LND@Lip (n = 3).

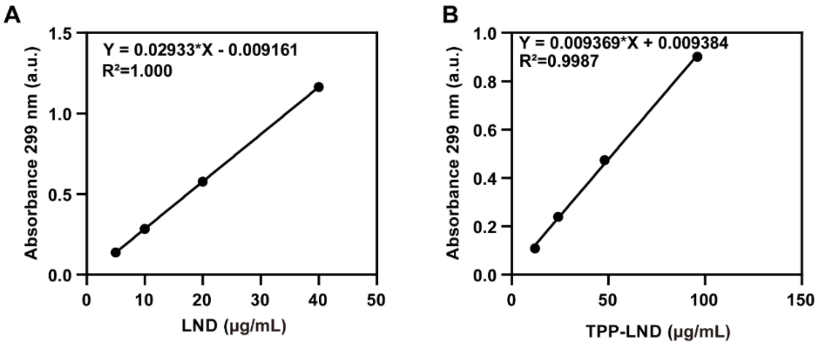

Figure S11. (A-B) Standard curves of LND (299 nm absorbance) and TPP-LND (299 nm absorbance) determined by UV-VIS spectroscopy.

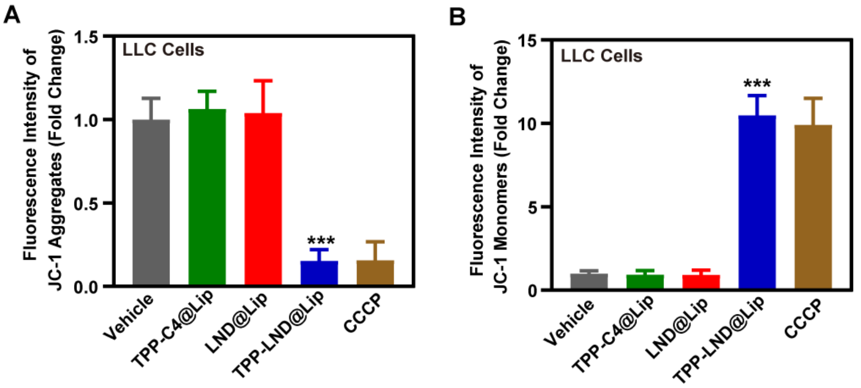

Figure S12. The normalized fluorescence intensity of JC-1 aggregates (red) and monomers (green) quantified by Image J from Fig. 3A. Data were presented as mean ± SD (n = 3). Statistical analysis was performed via the two-tail Student's *t*-test. * $p < 0.05$, ** $p < 0.01$, and *** $p < 0.001$.

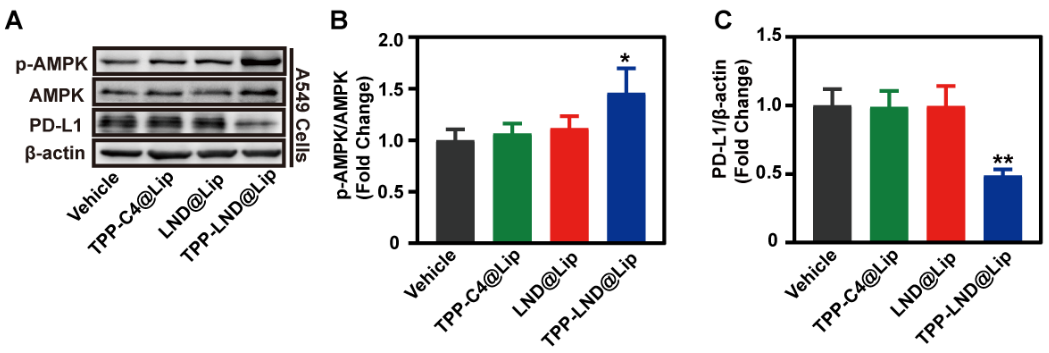

Figure S13. Expression of PD-L1, p-AMPK, and AMPK protein in A549 cells after different treatments for 24h detected by Western Blot assay and quantification by ImageJ. Data were presented as mean ± SD (n = 3). Statistical analysis was performed via the two-tail Student's *t*-test. * $p < 0.05$, ** $p < 0.01$, and *** $p < 0.001$.

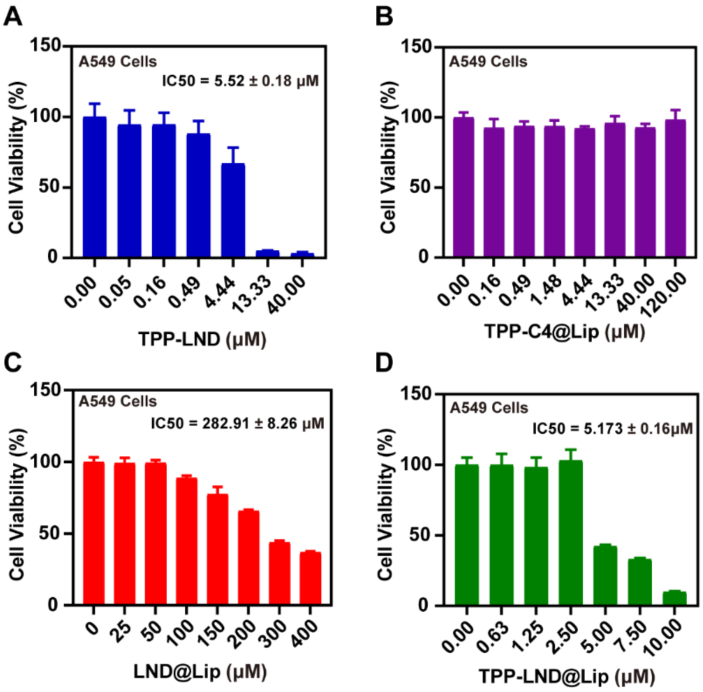

Figure S14. Cytotoxicity of A549 cells detected by CCK-8 assay after treatment with TPP-LND, TPP-C4@Lip, LND@Lip, or TPP-LND@Lip for 24 h. Data were presented as mean ± SD (n = 3).

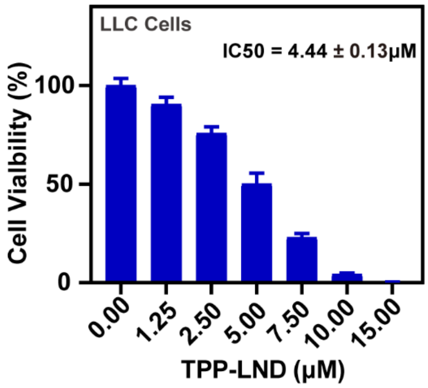

Figure S15. Cytotoxicity of LLC cells treated with different doses of TPP-LND for 24 h detected by CCK-8 assay. Data were presented as mean ± SD (n = 3).

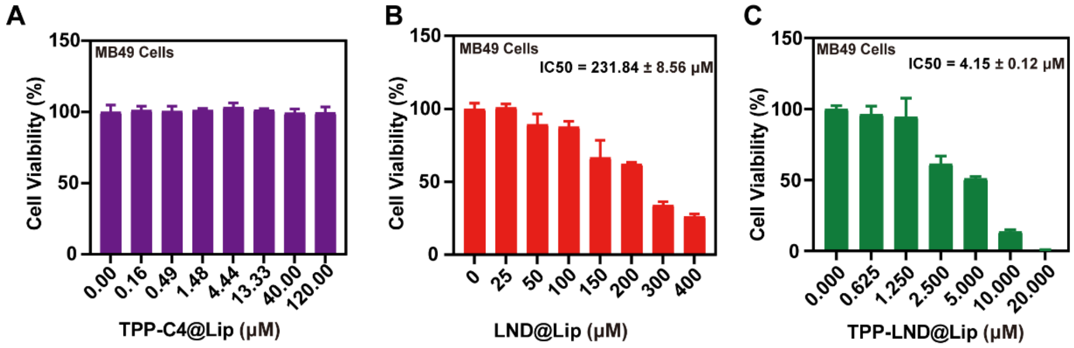

Figure S16. Cytotoxicity of MB49 cells detected by CCK-8 assay after treatments with TPP-C4@Lip, LND@Lip, or TPP-LND@Lip for 24 h. Data were presented as mean ± SD (n = 3).

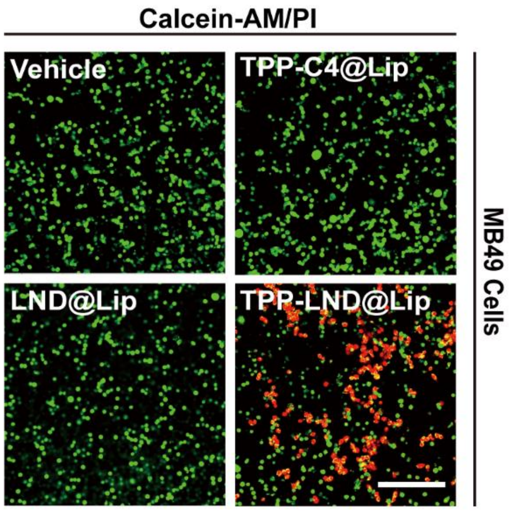

Figure S17. Live/dead cell staining assay of MB49 cells after TPP-C4@Lip, LND@Lip, or TPP-LND@Lip treatment, scale bar = 200 μm.

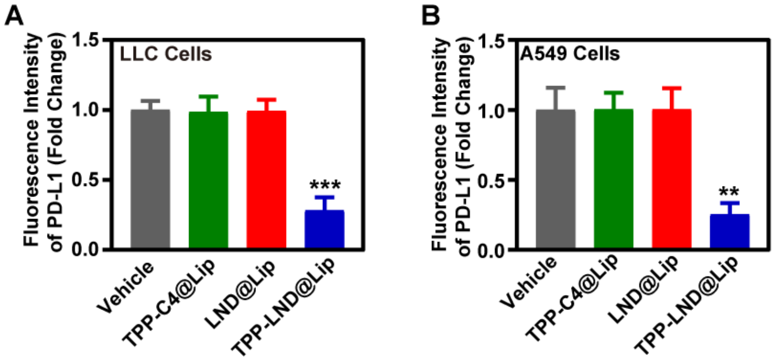

Figure S18. (A) The normalized fluorescence intensity of PD-L1 protein in LLC cells quantified by Image J from Fig. 4D. (B) The normalized fluorescence intensity of PD-L1 protein in A549 cells quantified by Image J from Fig. 4E. Data were presented as mean ± SD (n = 3). Statistical analysis was performed via the two-tail Student's *t*-test. * $p < 0.05$, ** $p < 0.01$, and *** $p < 0.001$.

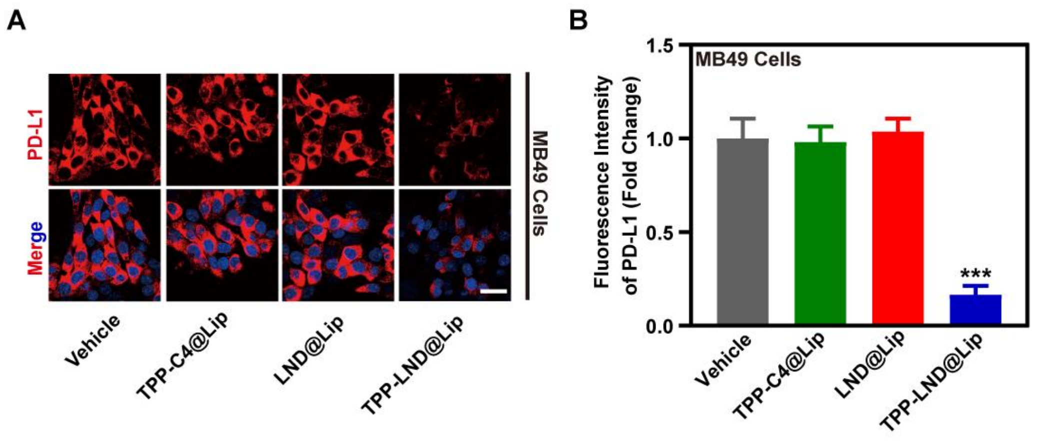

Figure S19. Representative CLSM images and quantification of PD-L1 immunofluorescence staining in MB49 cells after 3 μM TPP-C4@Lip, LND@Lip, or TPP-LND@Lip treatment, scale bar = 50 μm.

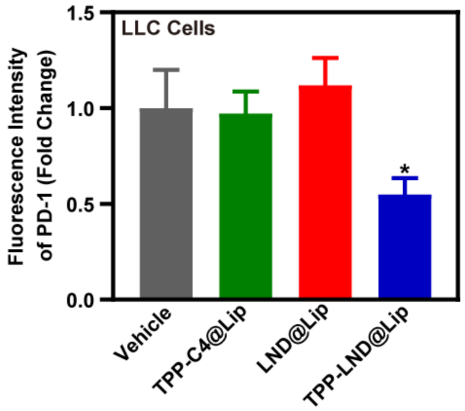

Figure S20. The normalized fluorescence intensity of PD-1 quantified by Image J from Fig. 4F. Data were presented as mean ± SD (n = 3). Statistical analysis was performed via the two-tail Student's *t*-test. * $p < 0.05$, ** $p < 0.01$, and *** $p < 0.001$.

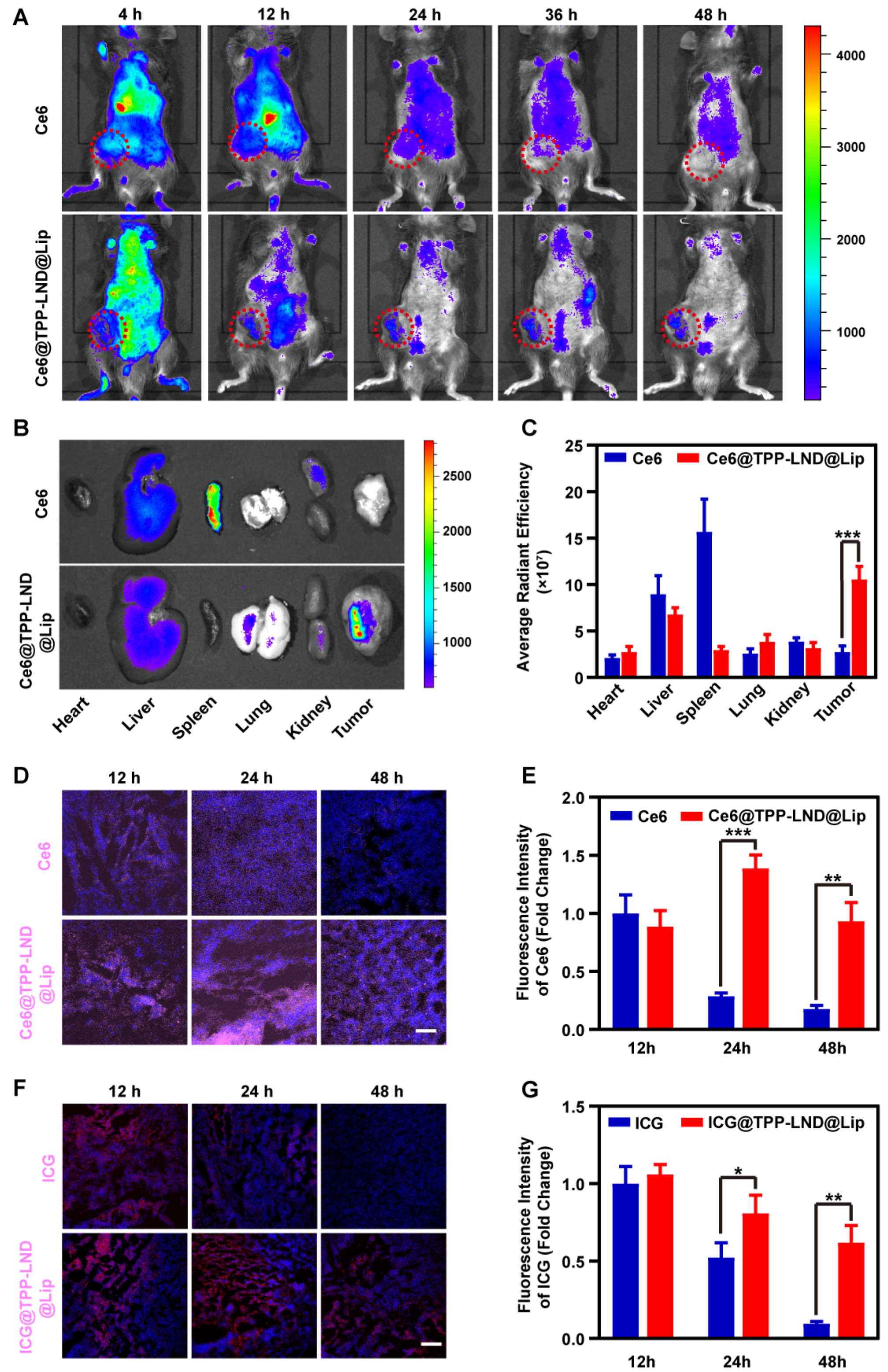

Figure S21. Evaluation of tumor targeting ability of TPP-LND@Lip *in vivo*. (A) Real-time NIR fluorescence images of LLC tumor-bearing mice after intravenous injection of Ce6 or Ce6@TPP-LND@Lip at different points of time. (B) NIR fluorescence images of main organs and tumor from LLC tumor-bearing mice after treatment with

Ce6 or Ce6@TPP-LND@Lip for 48 h. (C) Quantification of the average fluorescence efficiency of Ce6 from part B. (D) Representative fluorescence images of tumor tissues at preset time points (12 h, 24 h, and 48 h) after injection of Ce6 or Ce6@TPP-LND@Lip, scale bar = 100 μm. (E) The normalized fluorescence intensity of Ce6 quantified by Image J from part D. (F) Representative fluorescence images of tumor tissues at preset time points (12 h, 24 h, and 48 h) after injection of ICG or ICG@TPP-LND@Lip, scale bar = 100 μm. (G) The normalized fluorescence intensity of ICG quantified by Image J from part F. Data were presented as mean ± SD (n = 3). Statistical analysis was performed via the two-tail Student's *t*-test. * $p < 0.05$, ** $p < 0.01$, and *** $p < 0.001$.

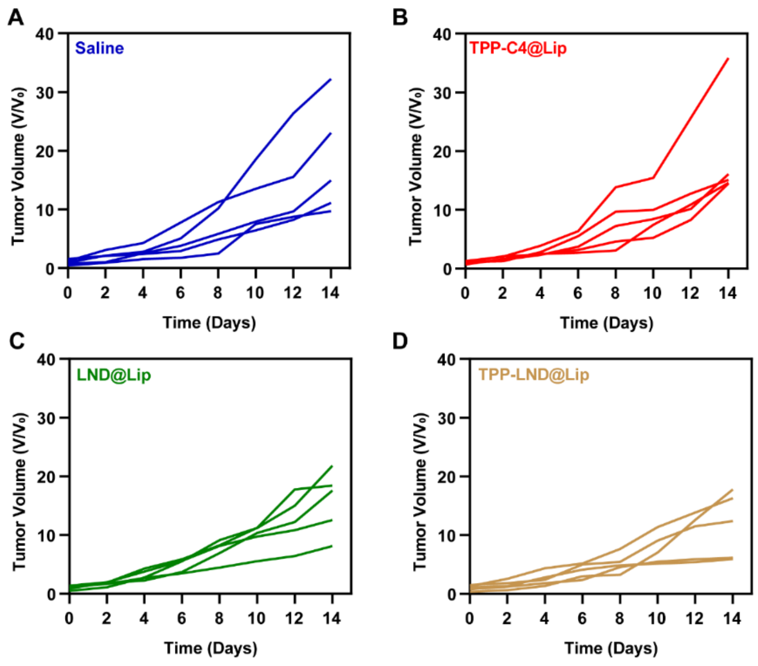

Figure S22. The antitumor effect of various treatments in the LLC tumor-bearing mice. The volume of LLC tumors after following treatments (n = 5). (A) Saline; (B) TPP-C4@Lip; (C) LND@Lip; (D) TPP-LND@Lip.

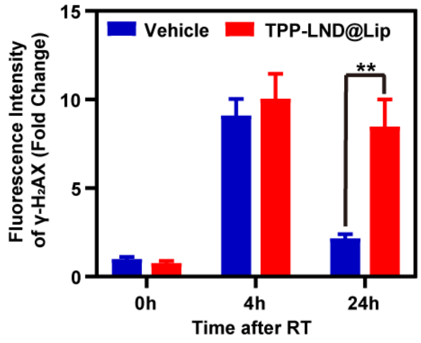

Figure S23. The normalized fluorescence intensity of γ-H_2AX protein quantified by Image J from Fig. 6D. Data were presented as mean ± SD (n = 3). Statistical analysis was performed via the two-tail Student's *t*-test. * $p < 0.05$, ** $p < 0.01$, and *** $p < 0.001$.

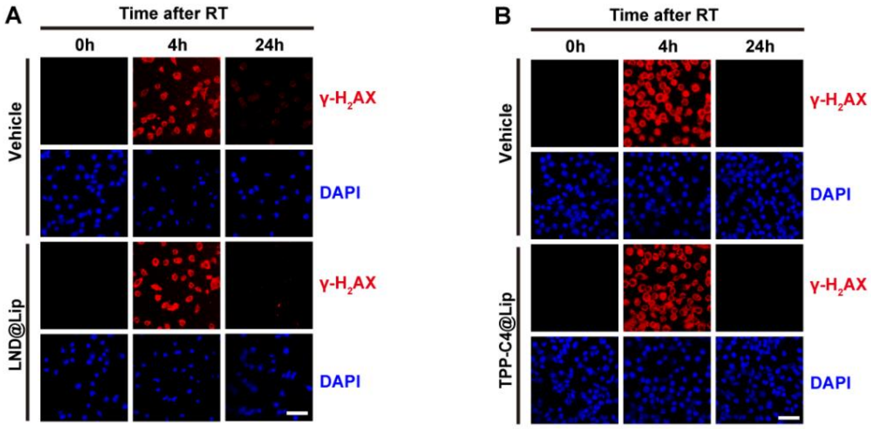

Figure S24. Representative fluorescence images of γ-H_2AX in LLC cells with radiotherapy (RT, 6 Gy) and LND@Lip or TPP-C4@Lip (3 μM), scale bar = 50 μm.

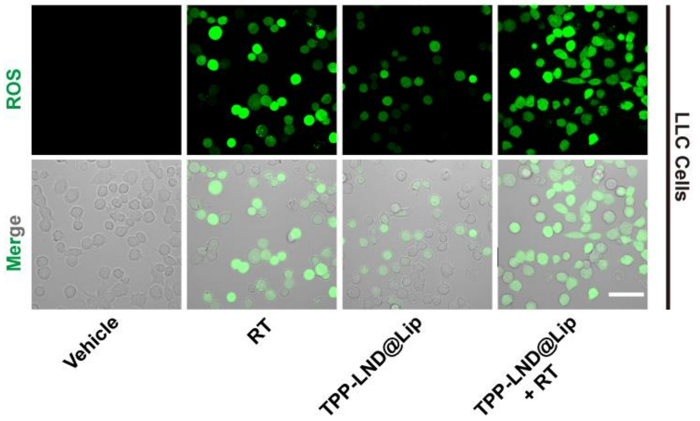

Figure S25. Representative images of ROS generation by DCFH-DA in LLC cells after different treatments, scale bar = 50 μm.

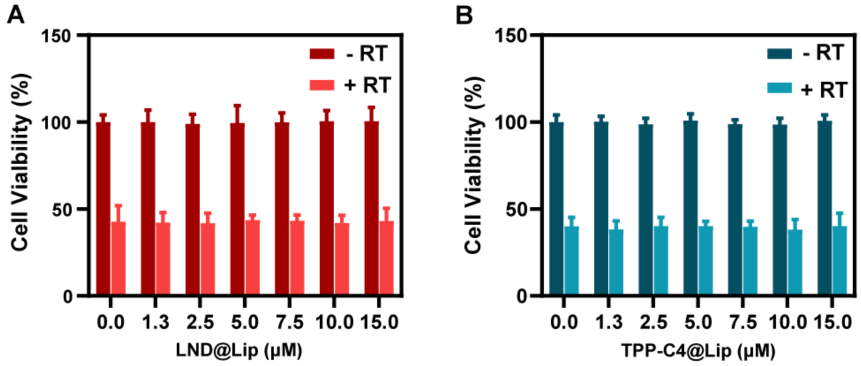

Figure S26. Cytotoxicity of LLC cells by CCK-8 assay after treatment with different concentrations of LND@Lip or TPP-C4@Lip in the presence or absence of RT (6 Gy). Data were presented as mean ± SD (n = 3).

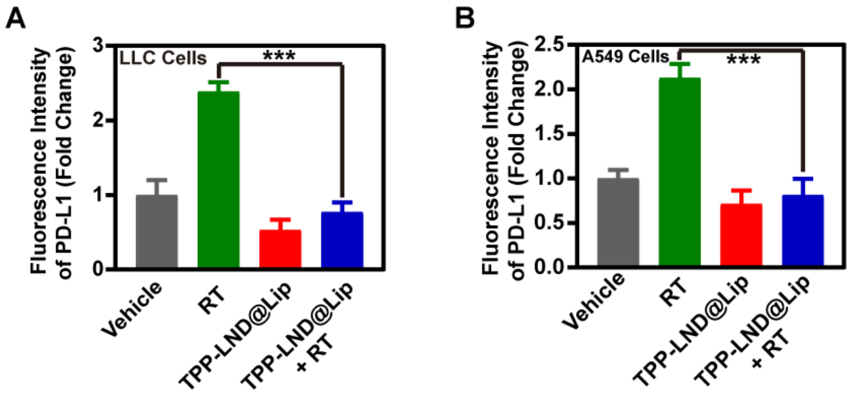

Figure S27. (A) The normalized fluorescence intensity of PD-L1 quantified by Image J from Fig. 7G. (B) The normalized fluorescence intensity of PD-L1 quantified by Image J from Fig. 7H. Data were presented as mean ± SD (n = 3). Statistical analysis was performed via the two-tail Student's *t*-test. * $p < 0.05$, ** $p < 0.01$, and *** $p < 0.001$.

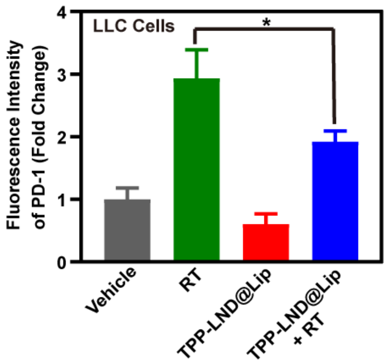

Figure S28. The normalized fluorescence intensity of PD-1 quantified by Image J from Fig. 7I. Data were presented as mean ± SD (n = 3). Statistical analysis was performed via the two-tail Student's *t*-test. * $p < 0.05$, ** $p < 0.01$, and *** $p < 0.001$.

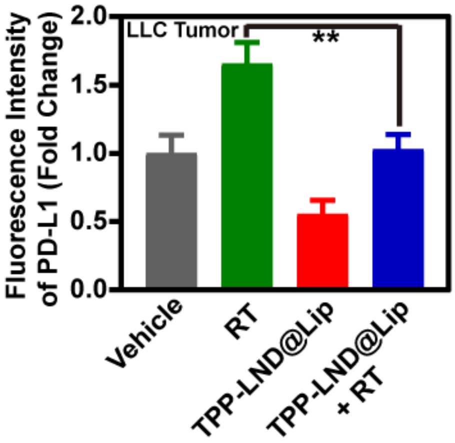

Figure S29. The normalized fluorescence intensity of PD-L1 quantified by Image J from Fig. 7L. Data were presented as mean ± SD (n = 3). Statistical analysis was performed via the two-tail Student's *t*-test. * $p < 0.05$, ** $p < 0.01$, and *** $p < 0.001$.

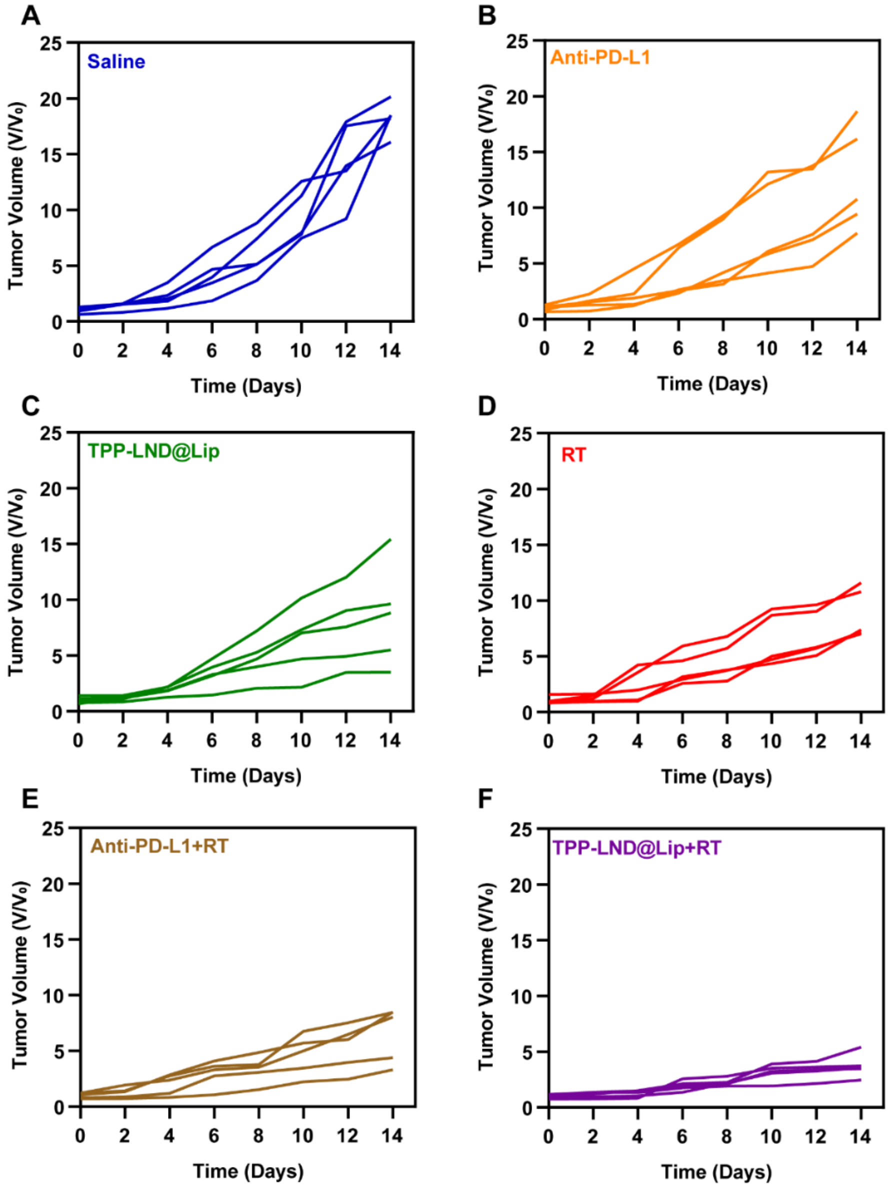

Figure S30. The antitumor effect of TPP-LND@Lip mediated radiotherapy in LLC tumor-bearing mice. (A-F) The volume curve of LLC tumors after following treatments (n = 5). (A) Saline; (B) PD-L1 antibody; (C) TPP-LND@Lip; (D) Radiotherapy; (E) PD-L1 antibody combined with radiotherapy; (F) TPP-LND@Lip combined with radiotherapy.

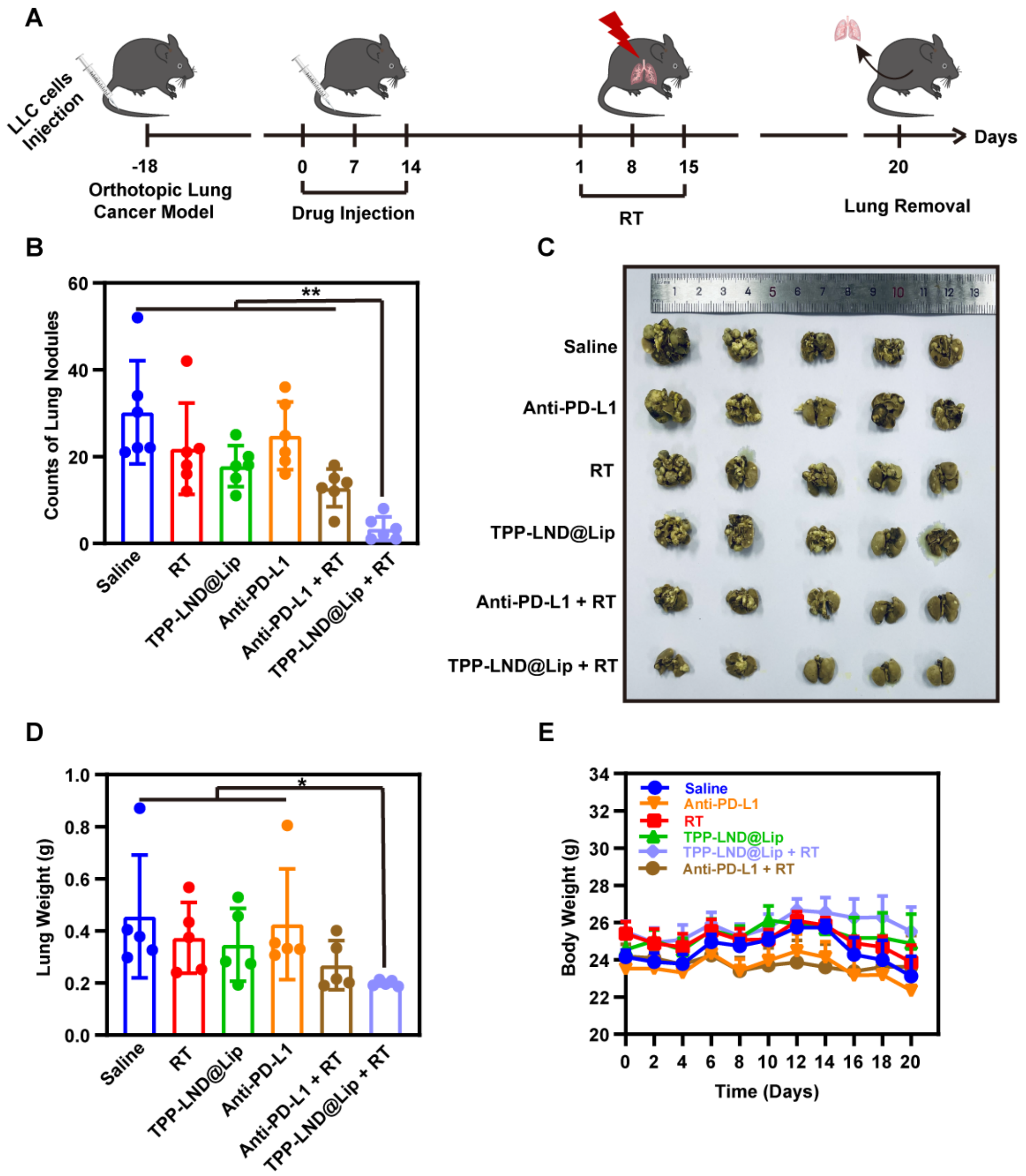

Figure S31. The antitumor effect of TPP-LND@Lip mediated radiotherapy on the orthotopic lung cancer model. (A) Schematic diagram of LLC tumor treatment process. (B) Counts of lung nodules after different treatments during 20 days (n = 5). (C) Photographs of excised lungs on Day 20 after different treatments (n = 5). (D) Weight of excised lungs on Day 20 (n = 5). (E) Bodyweight changes of lung tumor-bearing mice during 20 days (n = 5). Data were presented as mean ± SD. Statistical analysis was performed via the two-tail Student's *t*-test. * $p < 0.05$, ** $p < 0.01$, and *** $p < 0.001$.

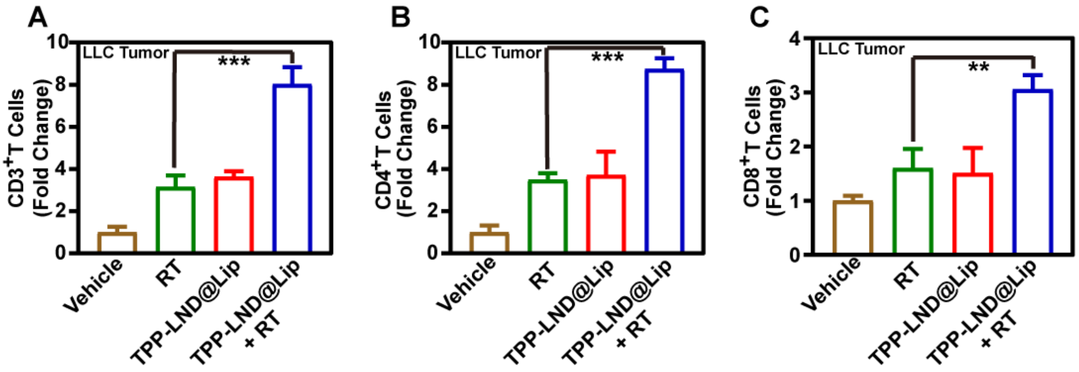

Figure S32. The normalized fluorescence intensity of $CD3^+$, $CD4^+$, and $CD8^+$ T cells quantified by Image J from Fig. 8F. Data were presented as mean ± SD (n = 3). Statistical analysis was performed via the two-tail Student's *t*-test. * $p < 0.05$, ** $p < 0.01$, and *** $p < 0.001$.

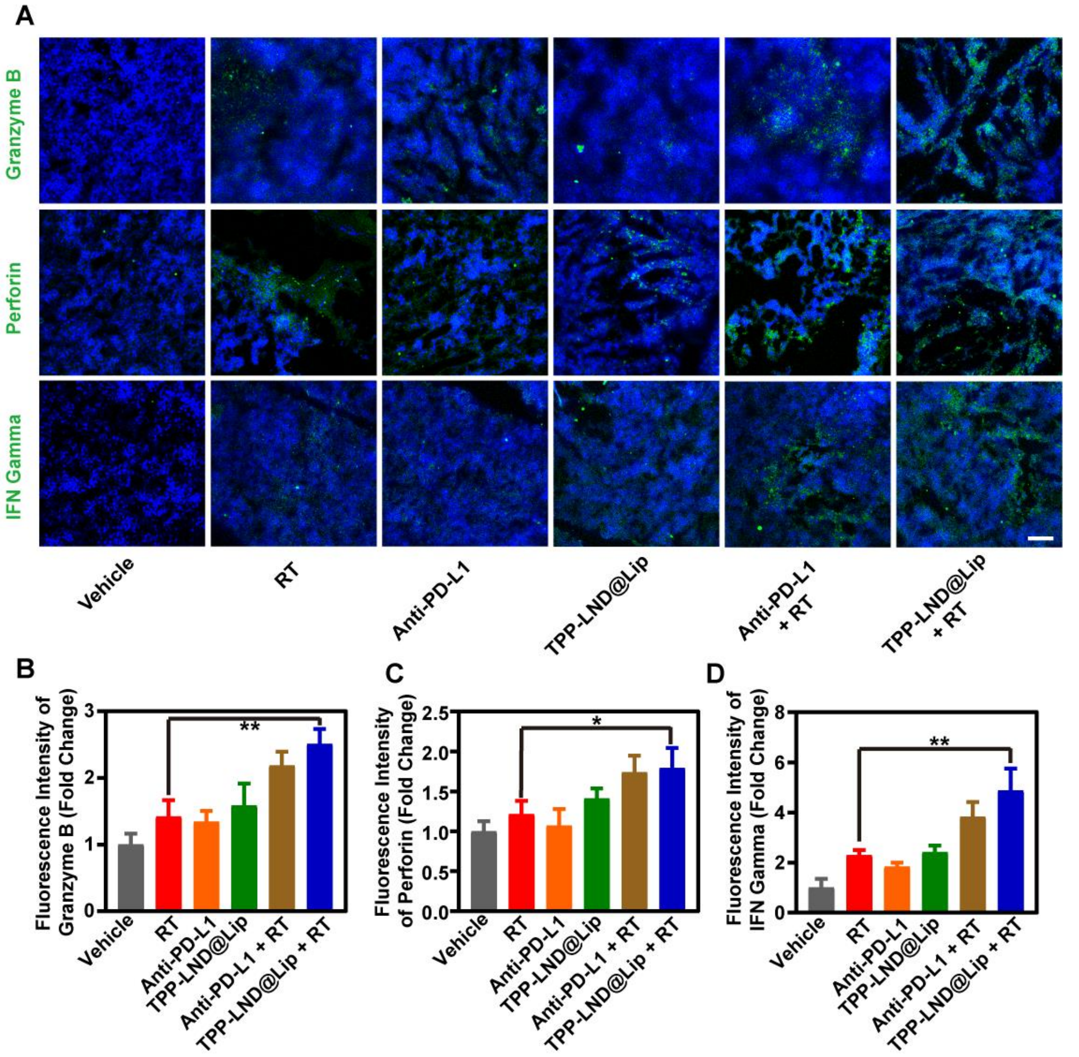

Figure S33. (A) Representative fluorescence images of Granzyme B, perforin, and IFN-γ in tumor slices after different treatments, scale bar = 100 μm. (B-D) Quantitative analysis of Granzyme B, perforin and IFN-γ in different groups by ImageJ. Data were presented as mean ± SD (n = 3). Statistical analysis was performed via the two-tail Student's *t*-test. * $p < 0.05$, ** $p < 0.01$, and *** $p < 0.001$.
